# Integrative Single-Cell Epigenomic Atlas Annotates the Regulatory Genome of the Adult Mouse Brain

**DOI:** 10.64898/2026.02.07.704075

**Published:** 2026-02-07

**Authors:** Zhaoning Wang, Songpeng Zu, Ethan J. Armand, Timothy H. Loe, Jonathan A. Rink, Wanying Wu, Yang Xie, Lei Chang, Chenxu Zhu, Nicholas D. Johnson, Jasper Lee, Jackson K. Willier, Silvia Cho, Stella Cao, Ariana S. Barcoma, Nora Emerson, Hanqing Liu, Kangli Wang, Zane A. Gibbs, Xiaomeng Gao, Sunan Xu, David Guo, Zhuowen Tu, Yang E. Li, Joseph R. Ecker, M. Margarita Behrens, Bing Ren

**Affiliations:** 1. Department of Cellular and Molecular Medicine, University of California, San Diego School of Medicine, La Jolla, CA; 2. Department of Genetics and Development, Columbia University Irving Medical Center, New York, NY; 3. Computational Neurobiology Laboratory, The Salk Institute for Biological Studies, La Jolla, CA; 4. Department of Genetics, Washington University School of Medicine, St. Louis, MO; 5. Department of Neurosurgery, Washington University School of Medicine, St. Louis, MO; 6. New York Genome Center, New York, NY; 7. Department of Systems and Computational Biomedicine, Weill Cornell Medicine, New York, NY; 8. Genomic Analysis Laboratory, The Salk Institute for Biological Studies, La Jolla, CA; 9. Junior Fellow, Faculty of Arts and Sciences, Harvard University, Boston, MA; 10. Department of Neurobiology, Harvard Medical School, Boston, MA; 11. Department of Cognitive Science, University of California, San Diego, La Jolla, CA; 12. Howard Hughes Medical Institute, The Salk Institute for Biological Studies, La Jolla, CA; 13. Department of Systems Biology, Columbia University Irving Medical Center, New York, NY; 14. Department of Biochemistry and Molecular Biophysics, Columbia University Irving Medical Center, New York, NY; 15. Lead contact

## Abstract

Histone modifications underpin the cell-type-specific gene regulatory networks that drive the remarkable cellular heterogeneity of the adult mammalian brain. Here, we profiled four histone modifications jointly with transcriptome in 2.5 million nuclei across multiple adult mouse brain regions. By integrating these data with existing maps of chromatin accessibility, DNA methylation, and 3D genome organization, we established a unified regulatory framework for over 100 brain cell subclasses. This integrative epigenomic atlas annotates 81% of the genome, defining distinct active, primed, and repressive states. Notably, active chromatin states marked by combinatorial histone modifications more precisely identify functional enhancers than chromatin accessibility alone, while Polycomb- and H3K9me3-mediated repression contributes prominently to cell-type-specific regulation. Finally, this multi-modal resource enables deep learning models to predict epigenomic features and gene expression from DNA sequences. This work provides a comprehensive annotation of the mouse brain regulatory genome and a framework for interpreting non-coding variation in complex tissues.

## INTRODUCTION

The mammalian brain is composed of an exceptionally diverse array of cell types that differ in developmental origin, molecular identity, anatomical distribution, and function^1,2^. Large-scale single-cell transcriptomic studies, including those from the BRAIN Initiative Cell Census Network (BICCN) and Allen Brain Atlas, have established a comprehensive taxonomy of brain cell types and revealed extensive cellular heterogeneity across brain regions and species^3–9^. These studies have provided a foundational framework for understanding brain organization, but transcriptional profiles alone offer limited insight into the regulatory mechanisms that establish and maintain cell-type-specific gene expression programs^10–13^.

Gene regulation in all mammalian cells is governed by a multilayered epigenome architecture that includes chromatin accessibility, histone modifications, DNA methylation, and three-dimensional (3D) genome organization^14,15^. In the mammalian brain, the extraordinary diversity of cell types, coupled with their precise spatial organization and developmental lineages, greatly amplifies regulatory complexity, necessitating coordinated interrogation of multiple epigenomic layers to resolve cell-type-specific regulatory mechanisms. Recent single-cell epigenomic studies have begun to map chromatin accessibility, DNA methylation and chromatin conformation at cell-type resolution in the mouse and human brain, substantially expanding catalogs of candidate *cis-*regulatory elements (cCREs) in the brain and revealing their cell-type-specific deployment^11,16–22^. However, histone modification landscapes, which represent a central regulatory layer, have remained poorly resolved at single-cell resolution across brain cell types, limiting mechanistic interpretation of regulatory element function and repression.

Histone modifications play essential roles in gene regulation by modulating the accessibility of regulatory elements to transcription machinery and regulating the communications between distal enhancers and target gene promoters. Acetylation and methylation of specific histone residues distinguish active, primed, poised, and repressed regulatory states, and these signatures have been extensively characterized in bulk tissues and cultured cell lines^23–27^. For example, acetylation of lysine 27 on histone H3 (H3K27ac) marks both active promoters and enhancers, tri-methylation of lysine 27 on histone H3 (H3K27me3) marks the facultative heterochromatin regions, mono-methylation of lysine 4 on histone H3 (H3K4me1) is associated with both active and primed enhancers, and tri-methylation of lysine 9 on histone H3 (H3K9me3) demarcates constitutive heterochromatin^28–32^. In complex tissues such as the brain, however, bulk profiling obscures cell-type-specific chromatin states, while technical challenges have constrained the scale and scope of single-cell histone modification mapping^33^. As a result, how active and repressive histone programs are deployed across diverse brain cell types, and how they integrate with other epigenomic layers, remains largely unknown.

Recent methodological advances have enabled single-cell profiling of histone modifications, either as individual modalities or jointly with the transcriptome^34–43^. In particular, we previously developed Paired-Tag (parallel analysis of individual cells for RNA expression and DNA from targeted tagmentation by sequencing), a multi-omic strategy that simultaneously profiles nuclear transcriptome and histone modifications in single cells using combinatorial indexing^35^. This approach enables direct linkage of chromatin state and transcriptional output in heterogeneous tissues and is scalable to atlas-level studies. However, a comprehensive application of this strategy to systematically map histone modification landscapes across the adult brain has not yet been achieved.

In this study, we generated a large-scale single-cell epigenomic atlas of the adult mouse brain by jointly profiling transcription and histone modification in over 2.5 million nuclei sampled from multiple brain regions. By integrating these data with existing single-cell maps of chromatin accessibility, DNA methylation, and 3D genome organization^16,17^, and anchoring all modalities to a unified brain cell-type taxonomy, we construct a cell-type-resolved framework encompassing more than one hundred neuronal and non-neuronal subclasses of the adult mouse brain. This resource enables functional annotation of the regulatory genome, distinguishes active and repressive chromatin states, and provides a foundation for predictive modeling of gene regulation in the mammalian brain.

## RESULTS

### A large-scale single-cell atlas of histone modification landscapes in the mouse brain

**Figure 1.**
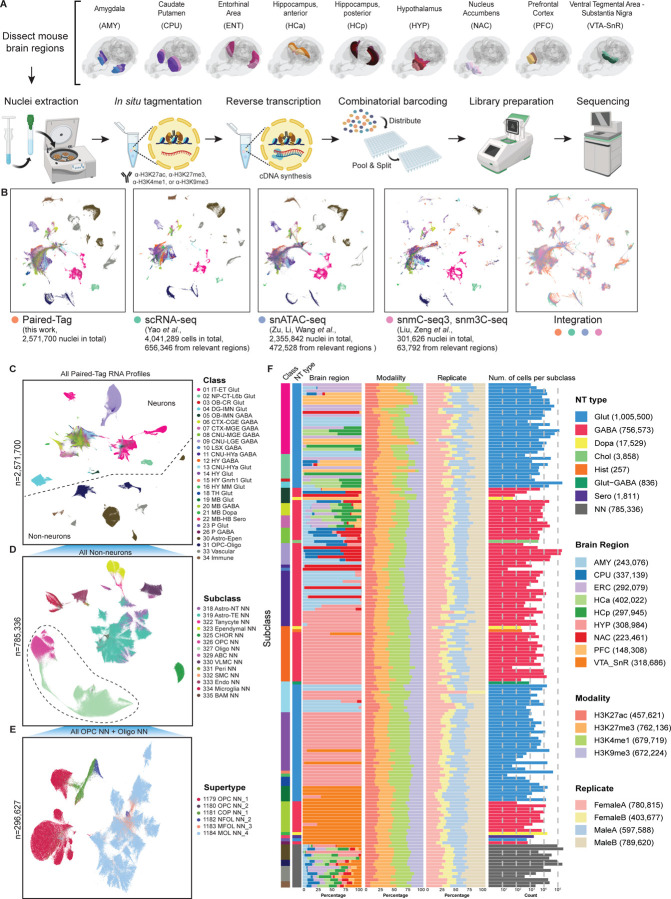
Single-cell co-analysis of gene expression and histone modifications in the adult mouse brain. A. Schematic overview of the Paired-Tag workflow used to jointly profile nuclear transcription and histone modifications in single cells from nine adult mouse brain regions. Brain regions analyzed include amygdala (AMY), caudate putamen (CPU), entorhinal area (ERC), hippocampus-anterior (HCa), hippocampus-posterior (HCp), hypothalamus (HYP), nucleus accumbens (NAC), prefrontal cortex (PFC), and ventral tegmental area - substantia nigra (VTA-SnR). For each region, two biological replicates were generated for both male and female mice by pooling the same region from 2 to 8 animals. Brain region schematics were generated using coordinates from the Allen Mouse Brain Common Coordinate Framework (CCF) v3^46^. B. Integration and joint embedding of Paired-Tag RNA profiles with reference scRNA-seq, snATAC-seq, and snmC-seq3 datasets generated from matched brain regions, enabling unified cell-type annotation. C. Uniform manifold approximation project (UMAP) embedding of all single-nucleus Paired-Tag RNA profiles analyzed in this study, colored by class labels. D. UMAP visualization of reclustered non-neuronal cells, including the following classes: 30 Astro-Epen, 31 OPC-Oligo, 33 Vascular, and 34 Immune, colored by subclass labels. E. UMAP visualization of single nucleus Paired-Tag RNA profiles from subclasses 326 OPC NN and 327 Oligo NN, showing progressive differentiation from oligodendrocyte precursor cells (OPCs) to mature oligodendrocytes at supertype resolution. F. Summary bar plots showing, from left to right, class labels, major neurotransmitter (NT) types, major brain region distribution of cells categorized, histone modification modality co-profiled with single nucleus RNA-seq profiles, biological replicates composition, and the number of nuclei for each subclass present in the dataset.

To generate a comprehensive, cell-type-resolved map of histone modification landscapes in the adult brain, we applied Paired-Tag to jointly profile transcription and chromatin features in single nuclei collected from nine anatomically and functionally distinct mouse brain regions, including amygdala (AMY), caudate putamen (CPU), entorhinal area (ERC), hippocampus-anterior (HCa), hippocampus-posterior (HCp), hypothalamus (HYP), nucleus accumbens (NAC), prefrontal cortex (PFC, which includes dorsal anterior cingulate cortex, prelimbic and infralimbic regions), and ventral tegmental area - substantia nigra (VTA-SnR). These brain regions were selected due to their critical roles in cognition, memory and known vulnerabilities in neurodegenerative diseases^44,45^ (Figure 1A). Dissections were performed on 600-um-thick coronal brain slices guided by the Allen Brain Reference Atlas^46^ (Figure S1A–C). For each sex, two biological replicates of samples were generated, each consisting of pooled dissections of the same brain region from 2–8 individual brains.

We focused on four major histone modifications: H3K27ac, H3K4me1, H3K27me3, and H3K9me3, the combinations of which mark the active, primed, and repressive regulatory states of chromatin. We performed more than 144 Paired-Tag reactions to profile four histone modifications jointly with the nuclear transcriptome using a modified Paired-Tag protocol with ultra-high throughput (Figure 1A) (Methods). In this improved protocol, we designed new barcodes in the adapter sequences loaded to protein A-Tn5 and in reverse transcription primers, as well as ligation barcodes used for combinatorial barcoding, which significantly expanded the barcoding space of PairedTag compared with the original workflow (see Methods and Table S1). Using specificity-validated antibodies, the resulting single-cell transcriptomic and epigenomic profiles were examined and quality-controlled using various metrics, including a stringent multiplet detection and removal procedure guided by experimentally determined multiplet rates, suggesting high quality and complexity of the dataset (Figure S1D; Figures S2A, B; and Methods). Altogether, these efforts yielded over 2.5 million single-nucleus RNA-histone modification joint profiles, representing one of the largest single-cell epigenomic datasets for a complex mammalian tissue to date (Table S2).

### Cell-type-resolved integration of transcriptomic and epigenomic modalities

To assign epigenomic profiles to defined brain cell types, we used the transcriptomic modality of Paired-Tag to anchor single-nucleus profiles to the mouse whole-brain cell-type taxonomy of the Allen Brain Cell Atlas^6^. For snRNA-seq profiles derived from Paired-Tag, we performed five rounds of iterative clustering using SnapATAC2^47^. For L1-level clustering, we used the differentially expressed genes identified from the Allen Brain Cell Atlas as variable features to identify major cell clusters^6^. In the subsequent re-clustering, top variable features from Paired-Tag snRNA-seq profiles were identified from the parental cluster (Methods). This resulted in a total of 4,302 L5-level cell clusters from the Paired-Tag snRNA-seq. For cell annotation, we performed integration analysis with the Allen Brain Cell Atlas as a reference, and annotated each L5-level cluster of Paired-Tag snRNA-seq profiles to the supertype resolution, and mapped their subclass and class-level classification based on the hierarchical relationship defined in the Allen Brain Institute’s whole mouse brain transcriptomic cell-type taxonomy^6^ (Figure 1B). To ensure high-quality cell type annotation, we performed integration for neurons and non-neurons separately, where neuronal Paired-Tag snRNA-seq profiles from each brain region were integrated with reference scRNA-seq data generated from comparable brain regions, which resulted in excellent correspondence between datasets (Figure S2C). We also integrated other previous independent single-cell epigenomic datasets from comparable brain regions, confirming robust cell type classification and providing the largest and most comprehensive single-cell atlas of the mammalian brain spanning multiple modalities, integrating RNA, chromatin accessibility, four histone modifications, DNA methylation, and 3D genome interactions to provide a unified, cell-type-resolved epigenomic framework for the adult mouse brain^16,17^ (Figure 1B).

The cell type annotations at various taxonomy levels were nicely resolved using the RNA modality of the Paired-Tag data by dimensional reduction and visualization using uniform manifold approximation and projection (UMAP). Various classes of brain cell types were resolved in the UMAP containing the entire Paired-Tag snRNA-seq dataset with over 2.5 million single cell profiles, and subsequent reclustering on over 785K non-neurons and over 296K cells from oligodendrocyte precursor cells (OPC)/oligodendrocytes (Oligo) subclasses further revealed their heterogeneity at subclass and supertype resolutions, respectively (Figures 1C–E). In this work, we provide single-cell RNA-histone modification joint profiles for 27 out of 34 major cell classes, 164 out of 338 subclasses, and 519 out of 1201 supertypes of the entire mouse brain cell taxonomy^6^. The cell types not represented in this study primarily originate from brain regions such as the cerebellum, certain midbrain areas, and the olfactory bulb, which were not sampled.

We also applied SnapATAC2 to perform histone mark-based dimensional reduction for single-cell Paired-Tag histone modification profiles using 5 kilobase (kb) genomic bin features (for H3K27ac and H3K4me1) or 50 kb genomic bin features (for H3K27me3 and H3K9me3) (Figure S2D). Supporting our previous results, we found that clustering and dimensional reduction based on single-cell profiles of active chromatin mark H3K27ac and active/primed enhancer mark H3K4me1 were able to nicely resolve different cell classes, as determined by RNA-based cell annotation^35^, with normalized mutual information (NMI) value achieving 0.6298 and 0.6736 and k-nearest neighbor (kNN) label transfer accuracy of 0.8519 and 0.8707 for H3K27ac and H3K4me1, respectively (Figure S2D). For the repressive histone marks analyzed in this study, facultative heterochromatin mark H3K27me3 showed better clustering resolution (NMI = 0.6669, kNN accuracy = 0.8528) than the constitutive heterochromatin mark H3K9me3 (NMI = 0.5269, kNN = 0.7472), where neuronal and non-neuronal cells were clearly clustered separately, but different neuronal cell classes were not resolved (Figure S2D). These observations highlight that different histone modifications operate at distinct regulatory levels to define brain cell identity, with H3K9me3 primarily separates major neuronal and non-neuronal classes, whereas H3K27me3 and active chromatin marks (H3K27ac and H3K4me1) fine-tune subclass-specific gene regulatory programs. Since Paired-Tag is a single-cell multiomic technology with RNA being co-profiled and used for cell annotation, these results also highlight the unique advantage of Paired-Tag over other single-modality single-cell epigenomic technologies in determining cell-type-specific epigenomic features from complex tissues.

Consistent with previous studies, we found that most neuronal subclasses and some non-neuronal cell subclasses showed strong regional specificity^6,17^ (Figure 1F, Figure S3 and Table S2). Notably, 117 of 164 brain cell subclasses showed pronounced regional enrichment, with more than 75% of cells within the subclass originating from one dominant brain region. When examining individual brain regions, we found that the hypothalamus, ventral tegmental area - substantia nigra, and amygdala contained the largest number of region-specific subclasses, highlighting a high degree of region-specific cellular heterogeneity in these brain areas (Figure S3). Furthermore, we compared the cellular composition of the anterior and posterior hippocampus, which were profiled separately using Paired-Tag. While most GABAergic neuronal and non-neuronal subclasses showed little regional specificity between these two anatomical subdivisions, several glutamatergic neuronal subclasses displayed marked spatial enrichment. Specifically, subclass 025 CA2-FC-IG Glut was enriched in the anterior hippocampus, whereas subclasses 023 SUB-ProS Glut and 033 NP SUB Glut were enriched in the posterior hippocampus (Figure S3), consistent with the known spatial organization of these hippocampal neuron populations^6,10^. Together, these data further suggest that distinct brain regions harbor specialized cell types and underlying gene programs that support region-specific functions.

### A comprehensive epigenome map of mouse brain cell types

**Figure 2.**
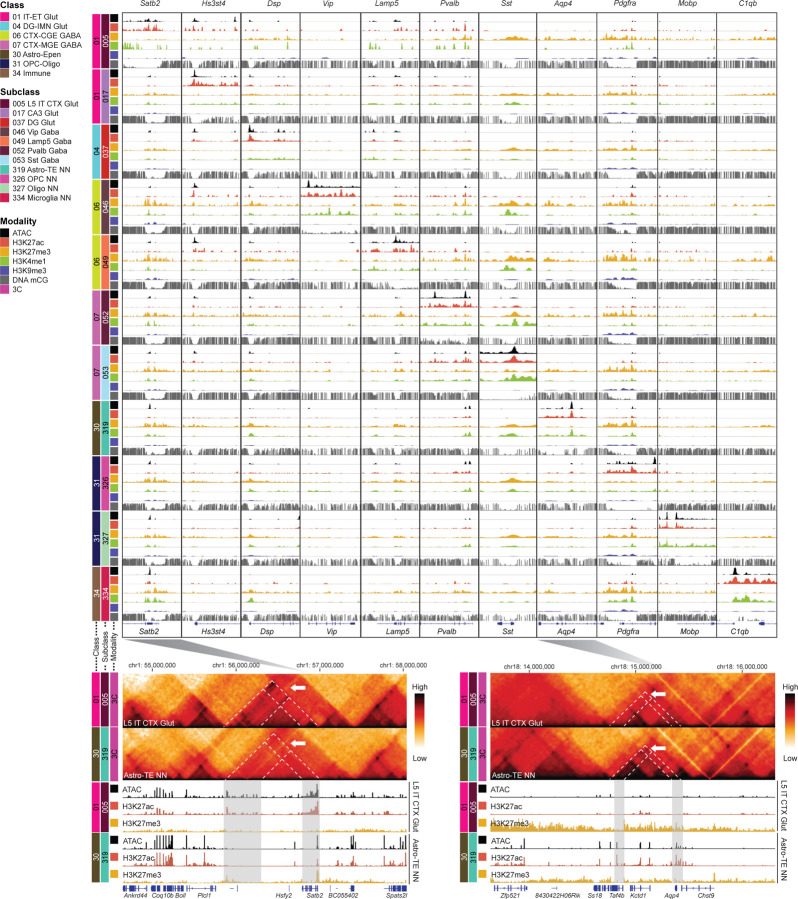
A comprehensive, cell-type-resolved epigenome map of the adult mouse brain. Genome browser views illustrating integrated epigenomic features across representative brain cell subclasses. (Top) Aggregated signals from snATAC-seq, H3K27ac Paired-Tag, H3K27me3 Paired-Tag, H3K4me1 Paired-Tag, H3K9me3 Paired-Tag, and DNA methylation at CG sites (mCG) for selected brain cell subclasses at cell-type-specific marker gene loci. These marker genes include: *Satb2*, *Hs3st4*, *Dsp*, *Vip*, *Lamp5*, *Pvalb*, *Sst*, *Aqp4*, *Pdgfra*, *Mobp*, and *C1qb*. (Bottom) Cell-type-specific chromatin contact maps showing enhancer-promoter interactions at the *Satb2* gene locus in 005 L5 IT CTX Glut, and at the *Aqp4* gene locus in 319 Astro-TE NN, highlighting coordinated activation of distal regulatory elements and target gene expression.

After aligning Paired-Tag data with other single cell epigenomic data using Allen Brain Cell Atlas as the common anchor, we depicted the most comprehensive epigenome atlas of the adult mouse brain, encompassing seven epigenetic modalities including chromatin accessibility, histone modification landscapes of H3K27ac, H3K27me3, H3K4me1 and H3K9me3, DNA methylation, and 3D chromatin interactions, significantly expanding our knowledge on top of the epigenetic modalities that have been characterized by previous studies as part of the BRAIN Initiative Cell Census Network (BICCN). A snapshot at certain cell-type-specific marker genes across selected subclasses of brain cell types revealed a high level of chromatin accessibility, enrichment of H3K27ac and H3K4me1 marks, depletion of H3K27me3 and H3K9me3 marks, and DNA hypomethylation, in a cell-type-specific manner, as hallmarks of gene activation (Figure 2, top). Further examination of genomic regions surrounding *Satb2* and *Aqp4*, marker genes specifically enriched in excitatory cortical neurons and astrocytes, respectively, revealed elevated chromatin contacts of proximal and distal regions with open and active chromatin marks in a cell-type-specific manner, suggesting the potential function of distal elements as enhancers through long-range chromatin looping (Figure 2, bottom). Together, this integrative epigenome map of the adult mouse brain provides a comprehensive atlas of epigenomic features that elucidates gene regulatory elements and mechanisms at the level of cell types. These epigenomic data have also been released through the CATlas database portal (https://catlas.org/catlas/amb-pt), allowing researchers to interactively explore the epigenomic landscapes of all the cell types characterized in this study (see Data availability).

### Chromatin state modeling functionally annotates the brain regulatory genome

**Figure 3.**
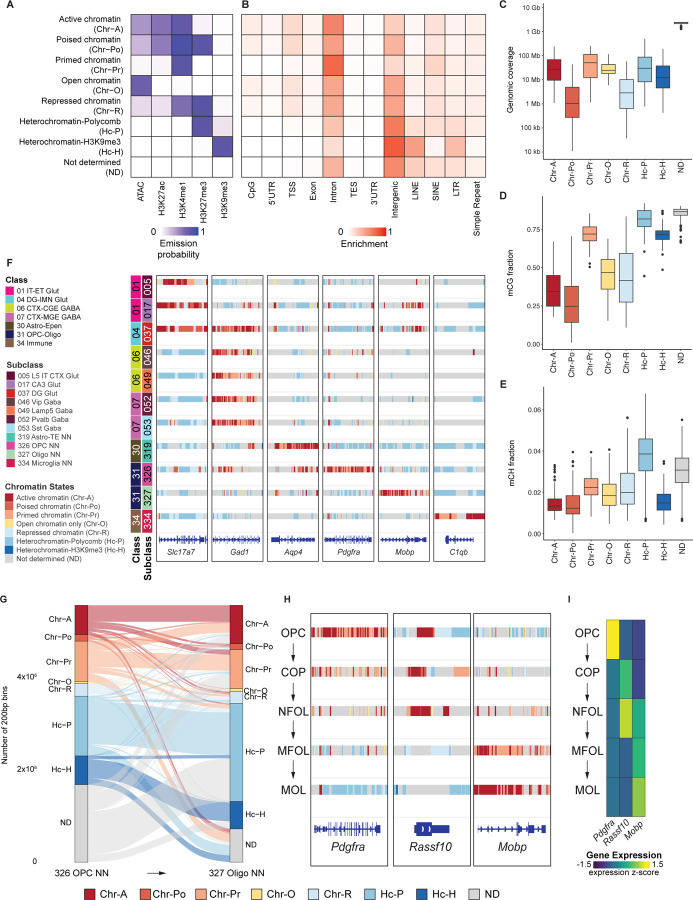
Chromatin state modeling functionally annotates the brain regulatory genome. A. Heatmap showing the emission probability of each epigenomic mark across the 8 chromatin states. B. Heatmap showing the relative enrichment of each chromatin state across different genomic annotations. C. Box plot showing the distribution of genomic coverage of each chromatin state across brain cell subclasses. D. Box plot showing fraction of DNA CG methylation (mCG) associated with each chromatin state across brain cell subclasses. E. Box plot showing fraction of DNA non-CG methylation (mCH) associated with each chromatin state across brain cell subclasses. F. Chromatin state landscapes at the *Slc17a7*, *Gad1*, *Aqp4*, *Pdgfra*, *Mobp*, and *C1qb* loci across selected brain cell subclasses, including 005 L5 IT CTX Glut, 017 CA3 Glut, 037 DG Glut, 046 Vip Gaba, 049 Lamp5 Gaba, 052 Pvalb Gaba, 053 Sst Gaba, 319 Astro-TE NN, 326 OPC NN, 327 Oligo NN, and 334 Microglia NN. G. Alluvial plot illustrating chromatin state transitions between 326 OPC NN and 327 Oligo NN. Chromatin regions that are annotated as ND for both 326 OPC NN and 327 Oligo NN subclasses are not shown. H. Chromatin state landscapes at the *Pdgfra*, *Rassf10*, and *Mobp* loci across selected cell supertypes reflecting cellular state transitions during the oligodendrocyte precursor cell (OPC) to oligodendrocyte differentiation process, including OPC, committed oligodendrocyte precursors (COP), newly formed oligodendrocytes (NFOL), myelin-forming oligodendrocytes (MFOL), and mature oligodendrocytes (MOL). I. Heatmap showing the relative expression of *Pdgfra*, *Rassf10*, and *Mobp* in OPC, COP, NFOL, MFOL and MOLs.

To integrate multiple epigenomic layers into a coherent regulatory framework, we used five epigenomic modalities (ATAC, H3K27ac, H3K27me3, H3K4me1, and H3K9me3) to combinatorially define chromatin states of the epigenome in each brain cell subclass. Using ChromHMM, we first generated models with 5 to 25 states and systematically compared their performance^48^ (Figures S4A–C). We found n=18 to be the minimum number of states to best capture the combinations of epigenetic marks analyzed in this study, as defined by a stringent quality control process requiring the median correlations of emission parameters > 99% and between-cluster variance over total variance (Between Sum of Square/Total Sum of Square) > 95%^27^ (Figures S4B, C). Next, we examined the deposition patterns of the five epigenetic marks on individual ChromHMM states across subclasses, and consolidated the 18 states defined by the model into 8 distinct chromatin states with a descriptive chromatin state name based on its similarity to known chromatin signatures, including active chromatin (Chr-A), poised chromatin (Chr-Po), primed chromatin (Chr-Pr), open chromatin (Chr-O), repressed chromatin (Chr-R), heterochromatin-Polycomb (Hc-P), heterochromatin-H3K9me3 (Hc-H), and not determined (ND) regions (Figures S4D, E; see Methods). These chromatin states showed distinct combinations of chromatin features and differential genomic annotation enrichments (Figures 3A, B). On average, ND regions cover a major proportion of the genome, whereas the Chr-Pr, Hc-P, Chr-A, and Hc-H states cover significantly more regulatory genome (>10 Mb) than the Chr-Po and Chr-R states (Figure 3C). Across brain cell subclasses, we found that Chr-Po and Chr-R states are the most variable, whereas ND regions with no detectable chromatin accessibility or histone modification signals showed the least variability between cell types (Figure S4F). In addition, we found Chr-A and Chr-Po contain the lowest levels of DNA methylation at both CpG (mCG) and non-CpG (mCH, where H can be A, C, or T) sites, whereas ND and Hc-P showed the highest mCG and mCH fractions (Figures 3D, E). On CpG islands, we found an inverse correlation of DNA methylation with H3K27me3 signal, consistent with previous reports that DNA methylation counteracts the H3K27me3 writer PRC2 at large CpG islands^49–52^ (Figures S4G). Hc-H also showed a considerable fraction of mCG, and the actual mCG fraction on *bona fide* Hc-H could be underestimated due to the open chromatin bias of Tn5 tagmentation that the DNA modality analysis of Paired-Tag (i.e., CUT&Tag) is based on^53^ (Figure 3D). Previous studies have shown that mCH preferentially accumulates in neurons compared with glia^54,55^. In line with this pattern, we found overall higher mCH fraction in all chromatin states in neuronal subclasses compared with non-neuronal subclasses, especially in Hc-P, suggesting differential involvement of mCH in neuronal gene regulation^16^ (Figures S4H, I). We further visualized chromatin state maps for representative neuronal and non-neuronal subclasses, highlighting the enrichment of active and primed chromatin states at cell-type-specific marker gene loci in the associated subclasses, whereas in subclasses where those marker genes are not expressed, Chr-R and Hc-P chromatin states are typically observed (Figure 3F). Cumulatively, our cell-type-resolved chromatin state map of the mouse brain annotated 29% of the genome to be Chr-A in any brain cell subclasses, 7% to be Chr-Po, 37% to be Chr-Pr, 24% to be Chr-O, 12% to be Chr-R, 40% to be Hc-P, and 18% to be Hc-H, providing functional chromatin state annotation to a total of 81% of the genome in the adult mouse brain (Figure S4J). This represents a substantial increase in the functional annotation of the adult mouse brain genome compared to our previous work, which utilized snATAC-seq and annotated 19% of the mouse genome as candidate *cis-*regulatory elements^17^.

### Dynamic chromatin state transitions during oligodendrocyte differentiation

In addition to characterizing chromatin states within static cell subclasses, our integrative brain epigenome map also enables the analysis of dynamic chromatin state transitions during the progression from OPCs to mature oligodendrocytes. By comparing chromatin states between subclass 326 OPC NN and subclass 327 Oligo NN, we observed extensive chromatin state conversions (Table S3). For example, of the 636,708 Chr-A regions identified in OPCs, 280,913 (44%) adopted a different chromatin state in oligodendrocytes (Figure 3G). The oligodendrocyte subclass contains 833,506 Chr-A regions in total, of which 355,795 (42%) are shared with OPCs. Newly acquired Chr-A regions in oligodendrocytes predominantly originate from Chr-P (207,191 regions, 25%) and no mark ND (177,255 regions, 21%) states in OPCs (Figure 3G). We next applied our ChromHMM model to all supertypes across the OPC-Oligo lineage (Figure 1E). We reconstructed the stepwise chromatin state transitions through each developmental stage of OPC to Oligo differentiation, including OPCs, committed oligodendrocyte precursors (COP), newly formed oligodendrocytes (NFOL), myelin-forming oligodendrocytes (MFOL), and mature oligodendrocytes (MOL)^6^ (Figure 3H), and examined their correlation with gene expression dynamics (Figure 3I). The *Pdgfra* locus, which encodes the canonical OPC surface marker platelet derived growth factor receptor alpha, is enriched for Chr-A and Chr-Pr states in OPCs^56,57^. These active chromatin signatures progressively diminish during differentiation, and most of the region adopts a Polycomb-repressed heterochromatin signature (Hc-P) in mature oligodendrocytes (Figure 3H, left). *Rassf10* encodes Ras association domain family member 10, a tumor suppressor that negatively regulates cell proliferation^58,59^. At this locus, we observed a core Chr-A region shared across OPC, COP, and NFOL stages, but the surrounding regions annotated as Chr-R and Hc-P in OPCs become resolved in COPs and NFOLs. This transition coincides with peak *Rassf10* expression (Figure 3I). As cells progress through the MFOL and MOL stages and activate the myelin gene program, the locus loses its active states and shifts to ND and repressive states, including Chr-R and Hc-P. These dynamics support a role for *Rassf10* in promoting cell cycle exit and lineage commitment during early oligodendrocyte differentiation (Figure 3H, middle). *Mobp* encodes myelin-associated oligodendrocyte basic protein, a structural component of the myelin sheath and a marker of mature oligodendrocytes^60^. Its locus is associated with multiple repressive chromatin states in OPCs that gradually resolve in COPs and NFOLs. In MFOLs, the region is largely in a primed Chr-Pr state with several Chr-A segments near the transcription start site, and in MOLs, the majority of the locus adopts a Chr-A signature (Figure 3H, right). Together, these findings indicate that dynamic chromatin state transitions during OPC-to-oligodendrocyte differentiation parallel the transcriptional activation and silencing of key lineage genes. This framework provides a detailed epigenomic map for uncovering regulatory elements that control this highly dynamic developmental process.

### Active chromatin states distinguish functional enhancers from open chromatin

**Figure 4.**
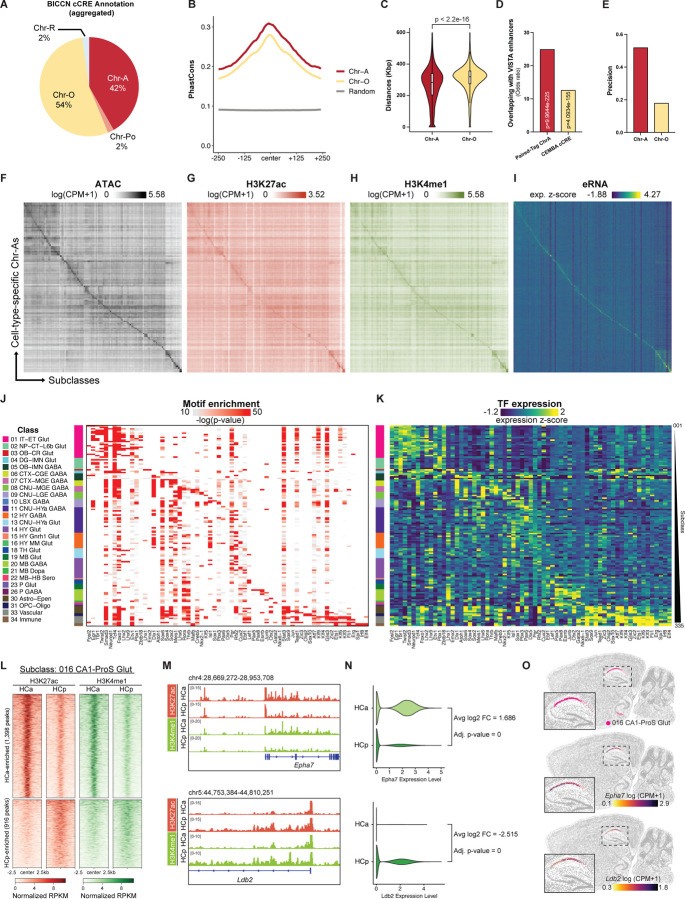
Functional annotation and epigenomic dissection of brain cCREs. A. Pie chart summarizing the overall chromatin state composition of cCREs aggregated across all brain cell subclasses. Brain cCREs were annotated by chromatin state separately within each subclass, and state assignments were then pooled across all subclasses to quantify the global distribution of chromatin states. Brain cCREs were previously defined using snATAC-seq^17^. B. Average PhastCons conservation scores of cCREs annotated as Chr-A (red) or Chr-O (yellow), compared with random genomic background (control) regions (grey). C. Violin plot showing the distribution of pairwise genomic distances between Chr-As or between Chr-Os within 1Mb genomic bins across brain cell subclasses. Statistical significance was assessed using the Wilcoxon rank-sum test. D. Bar plot showing the odds ratios for overlap between Chr-As identified by Paired-Tag (red) or cCREs identified using snATAC-seq (yellow) and validated enhancers with brain activities from the VISTA enhancer browser database. Statistical significance of overlap was evaluated using Fisher’s exact test. E. Bar plot showing the precision of Chr-A or Chr-O annotation in predicting validated cell-type-specific enhancers from the Allen Institute Enhancer (AiE) collection. Precision is defined as the fraction of predicted enhancer cCREs that were experimentally validated using Allen Institute AAV reporter assays. F. Heatmap showing chromatin accessibility (ATAC-seq signal) at cell-type-specific Chr-As across brain cell subclasses. G. Heatmap showing H3K27ac signal at cell-type-specific Chr-As across brain cell subclasses. H. Heatmap showing H3K4me1 signal at cell-type-specific Chr-As across brain cell subclasses. I. Heatmap showing enhancer RNA (eRNA) signal at cell-type-specific Chr-As across brain cell subclasses. J. Heatmap showing enrichment of cell-type-specific transcription factor binding motifs within Chr-As across brain cell subclasses. K. Heatmap showing the relative expression levels of corresponding cell-type-specific transcription factors across brain cell subclasses. L. Coverage heatmaps of H3K27ac and H3K4me1 signals at differentially enriched H3K27ac peaks between anterior (HCa) and posterior (HCp) hippocampus in subclass 016 CA1-ProS Glut neurons. M. Genome browser tracks showing differential H3K27ac and H3K4me1 signals in 016 CA1-ProS Glut neurons from HCa and HCp at *Epha7* (top) and *Ldb2* (bottom) genomic loci. N. Violin plots showing differential expression of *Epha7* (top) and *Ldb2* (bottom) in 016 CA1-ProS Glut neurons between HCa and HCp. Statistical significance was assessed using the Wilcoxon rank-sum test followed by Bonferroni correction. O. MERFISH analysis of the adult mouse brain showing (top) the spatial distribution of subclass 016 CA1-ProS Glut neurons in a sagittal brain section; (middle) anterior-enriched expression of *Epha7* in these neurons; and (bottom) posterior-enriched expression of *Ldb2*. MERFISH data were obtained from the Allen Brain Cell (ABC) Atlas web portal (https://brain-map.org/bkp/explore/abc-atlas).

Although chromatin accessibility is widely used to nominate candidate regulatory elements, accessibility alone does not distinguish functional enhancers from structurally open chromatin. Previously, we identified a total of 1 million candidate *cis*-regulatory elements (cCREs) in the adult mouse brain by profiling chromatin accessibility using snATAC-seq^17^. With cell-type-resolved chromatin state maps of the overlapping brain regions, we provide further functional annotation of these cCREs. First, examination of the chromatin states on the cCREs in each brain cell subclass revealed that an average of 42% of cCREs in each cell type carry Chr-A signatures, whereas 54% of the cCREs are Chr-Os with only chromatin accessibility but no other active histone marks (Figure 4A). Next, we sought to further characterize sequence features that distinguish Chr-A from Chr-O. In terms of sequence conservation, Chr-A regions showed a higher PhastCons conservation score than Chr-O^61^ (Figure 4B). In addition, we have previously constructed cell-type-resolved chromatin accessibility maps of the human brain^21^. Comparative epigenomic analysis further classified mouse brain cCREs into three groups: conserved (mouse cCRE has a conserved sequence with chromatin accessibility in the human brain), divergent (mouse cCRE has a conserved sequence but no detectable chromatin accessibility in the human brain), and non-conserved (mouse cCRE does not have a conserved sequence in the human genome). Compared with Chr-O regions, Chr-A of the mouse brain has a larger fraction of human conserved cCREs, and a smaller fraction of non-conserved cCREs, suggesting conserved sequence and function of mouse Chr-A compared to Chr-O (Figures S5A, B). Furthermore, in mouse cCREs that are differentially accessible across different subclasses of brain cell types, we found higher fractions of Chr-A than Chr-O, suggesting that Chr-As are preferentially enriched in cell-type-specific cCREs as potential cell-type-specific enhancers (Figure S5C). We also compared the pairwise genomic distances between regions annotated as Chr-A and Chr-O. Chr-A regions exhibited significantly shorter pairwise distances than Chr-O regions, indicating that Chr-A regions are more spatially clustered along the linear genome (Figure 4C). Notably, the Chr-A distribution displays a pronounced density at very short distances, which likely reflects groups of densely packed enhancer elements commonly known as “super-enhancers”^62^ (Figure 4C).

To directly examine the functional relevance of Chr-A and Chr-O regions, we cross-referenced a few validated brain enhancer catalogs. Using VISTA enhancer browser, we found that compared with cCREs defined by snATAC-seq, Paired-Tag-derived Chr-A regions better enrich enhancer sequences with validated activities in brain-related embryonic regions, including forebrain, midbrain, hindbrain and neural tube^63^ (Figure 4D). Additionally, for enhancers with high cell-type-specificity (“on-target” in label specificity) and can strongly activate reporter gene expression (“strong” in label brightness) that were previously characterized in the brain using AAV-enhancer reporters as part of the Allen Institute Enhancer (AiE) collection^64^ (Figure S5D), we examined the chromatin states of AiE in various cell subclasses, and found that Chr-A annotation on the enhancer sequence shows better precision, recall and specificity in predicting their cell-type-specific activities (Figure 4E; Figures S5E, F). Together, these data demonstrate that active chromatin (Chr-A) regions with combinatorial epigenetic marks (H3K27ac, H3K4me1, chromatin accessibility) better enrich functional enhancer elements than open chromatin (Chr-O) regions with only chromatin accessibility but no active chromatin marks.

To infer potential regulators of different chromatin states, we performed motif analysis on Chr-A, Chr-Pr, and Chr-O regions of each brain cell subclass. We detect the largest number of transcription factor (TF) binding motifs enriched in Chr-A regions. In contrast, there are fewer motifs enriched in Chr-Pr and Chr-O, especially in non-neuronal Chr-O regions (Figure S5G). In addition, we found lineage-specific transcription factor (TF) binding motifs to be most significantly enriched in Chr-A regions, such as various NeuroD and Neurogenin subfamily members of basic helix-loop-helix (bHLH) TFs in dentate gyrus glutamatergic neurons (subclass 037 DG Glut) and ETS family member TFs in microglia (subclass 334 Microglia NN) (Figure S5H). We also identified certain TF motifs enriched in Chr-Pr with much less significance, possibly regulating the primed but not yet activated gene programs in response to stress or cellular signaling (Figure S5H). Interestingly, we found that CTCF-related motifs are repeatedly discovered in Chr-O regions of almost every cell subclass we examined (Figure S5H, I). In contrast, motifs from the MEF2 transcription factor family, such as MEF2A, MEF2C, and MEF2D, were predominantly enriched in Chr-A regions in most subclasses (Figure S5J). These findings are consistent with the established role of MEF2 family members in regulating gene regulatory networks across diverse lineages, including both neuronal and non-neuronal cells^65,66^, and further support the notion that Chr-O regions are enriched for structural elements involved in chromatin looping and genome topology. Together, these data highlight differences in sequence conservation, functional outcomes, and motif enrichment between Chr-A and Chr-O, strongly implicating a regulatory role for active chromatin, as defined by Paired-Tag, as enhancer elements.

We next focused on distal cCREs annotated as Chr-A in at least one brain cell subclass and performed statistical testing to identify regions with cell-subclass-specific chromatin accessibility. This analysis nominated approximately 500K cell-type-specific Chr-A regions across more than one hundred brain cell subclasses (Figure 4F, Table S4). We then examined additional active chromatin signatures and found that H3K27ac and H3K4me1 signals were enriched in a cell-type-specific manner, closely paralleling their accessibility profiles (Figures 4G, H). Importantly, because the Paired-Tag RNA modality employs random primers during reverse transcription, we were able to detect cell-type-specific RNA signals from these regions that are likely enhancer RNAs, further supporting the enhancer activity of these Chr-A elements (Figure 4I). Together, these findings demonstrate that Chr-A regions represent a broad collection of candidate cell-type-specific enhancers in the adult mouse brain.

We next performed motif enrichment analysis to nominate key TF regulators from cell-type-specific Chr-As. This analysis revealed 839 TF motifs that are significantly enriched in the cell-type-specific Chr-As in at least one of the brain cell subclasses (Figure S5K, Table S5). We reasoned that, besides motif enrichment, TF gene expression also serves as an important criterion to nominate key cell-type-specific TFs. Therefore, we performed differential expression analysis using Paired-Tag RNA data to identify TF genes enriched within each brain cell subclass, and intersected that with TFs whose motifs also showed cell-type-specific enrichment. This integrative approach identified 65 TFs whose motifs and gene expression are both enriched in a subclass-specific manner, suggesting that they could serve as key TF regulators of subclass-specific gene regulatory networks (Figures 4J, K). Many of these TFs correspond to known lineage-specific regulators, supporting the biological coherence of the inferred key TF regulatory networks. For example, Tbr1 and NeuroD1 are selectively enriched in various glutamatergic neuronal subclasses and are known to regulate excitatory neuron identity and differentiation^67–69^. Lhx6 and Dlx1 show enrichment across various GABAergic subclasses, consistent with their central roles in interneuron specification, migration, and maturation^70–73^. In the oligodendrocyte lineage, Olig2 and Sox10 exhibit coordinated enrichment of expression and motif activity, in line with their established functions in oligodendrocyte specification and myelination^74–78^. Fli1 and Irf8 are enriched in vascular cells and/or immune lineages, consistent with their function in mesodermal/immune gene regulation^79–84^. Taken together, our analysis highlights the rich regulatory potential of cell-type-specific Chr-As, and pinpoints key transcriptional regulators responsible for cell-type-specific gene regulatory networks.

Since most neuronal cell subclasses exhibit strong anatomical regional specificity (Figure 1F, Figure S3), many of the above-identified subclass-specific epigenomic profiles are inherently specific to brain regions. A smaller subset of subclasses, however, is shared across multiple brain regions, raising the question of whether these common cell types also harbor region-specific regulatory programs. To address this, we focused on hippocampal glutamatergic subclasses present in both anterior and posterior hippocampus, including subclasses 016 CA1-ProS Glut, 017 CA3 Glut, and 037 DG Glut. Within each subclass, comparison of H3K27ac profiles between anterior and posterior hippocampus revealed distinct sets of region-enriched H3K27ac-marked active Chr-As (Figure 4L, Figures S5L, M), whose associated genes showed concordant region-enriched expression. For example, in subclass 016 CA1-ProS Glut, we identified stronger active chromatin signatures associated with *Epha7* gene in HCa and with *Ldb2* in HCp (Figure 4M). Paired-Tag scRNA-seq profiles confirmed the differential expression pattern of these genes between HCa and HCp (Figure 4N). Importantly, spatial transcriptomic analysis of the mouse brain using multiplexed error-robust fluorescence in situ hybridization (MERFISH) in a previous BICCN study independently validated the regional specificity of these genes within CA1-ProS Glut neurons (Figure 4O)^10^. Together, these results demonstrate that active chromatin landscapes encode region-specific regulatory specialization even within shared neuronal subclasses, enabling common cell types to adopt distinct gene regulatory programs across brain regions.

### Repressive chromatin programs refine lineage-specific gene regulation

**Figure 5.**
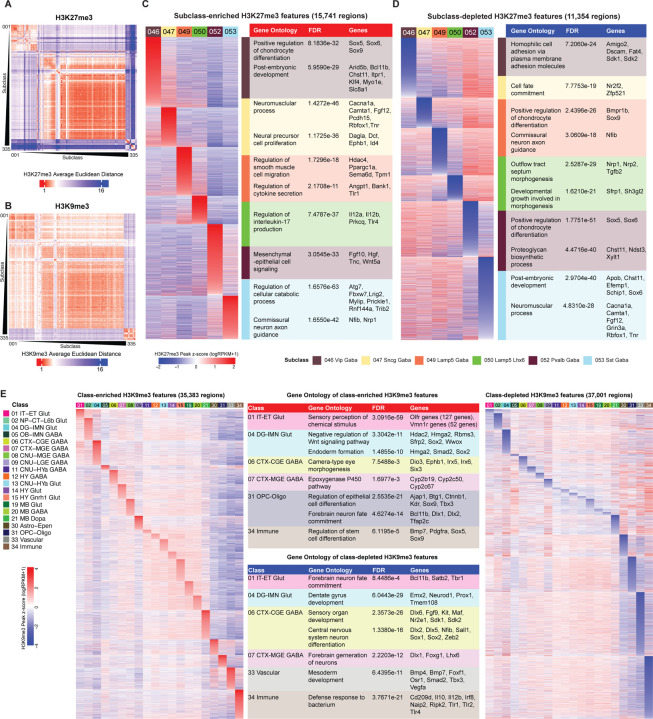
Cell-type-specific repressive histone modification features of the mouse brain. A. Heatmap showing the pairwise Euclidean distance between brain cell subclasses computed from low-dimensional embeddings of single-cell H3K27me3 Paired-Tag profiles. B. Heatmap showing the pairwise Euclidean distance between brain cell subclasses computed from low-dimensional embeddings of single-cell H3K9me3 Paired-Tag profiles. C. Left, heatmap showing normalized H3K27me3 signal over subclass-enriched H3K27me3 features in selected CGE- and MGE-derived GABAergic neuronal subclasses. Right, table showing Gene Ontology terms enriched among these features and their nearest gene targets. D. Left, heatmap showing normalized H3K27me3 signal over subclass-depleted H3K27me3 features in selected CGE- and MGE-derived GABAergic neuronal subclasses. Right, table showing Gene Ontology terms enriched among these features and their nearest gene targets. E. Left, heatmap showing normalized H3K9me3 signal over class-enriched H3K9me3 features across major brain cell classes. Right, table showing Gene Ontology terms enriched among these features and their nearest gene targets in selected cell classes. F. Left, heatmap showing normalized H3K9me3 signal over class-depleted H3K9me3 features across major brain cell classes. Right, table showing Gene Ontology terms enriched among these features and their nearest gene targets in selected cell classes.

In addition to active regulatory elements, cell identity in the brain is shaped by repressive chromatin programs. Analysis of H3K27me3 and H3K9me3 landscapes could reveal distinct modes of repression across brain cell types. H3K27me3, deposited by the Polycomb Repressive Complex 2 (PRC2), is a hallmark of facultative heterochromatin^85–87^. In contrast, H3K9me3 marks constitutive heterochromatin and is catalyzed by histone methyltransferases such as SUV39H1 (KMT1A), SUV39H2 (KMT1B), and SETDB1 (KMT1E), exhibiting genomic deposition patterns that are distinct from those of H3K27me3^88,89^. We first calculated the between-subclass average Euclidean distances of single-cell H3K27me3 and H3K9me3 profiles, and found that H3K27me3 showed a larger average Euclidean distance between subclasses than H3K9me3, especially when comparing subclasses from different brain cell classes (Figures 5A, B). This is consistent with our observation that single-cell H3K27me3 Paired-Tag DNA profiles showed better resolving power than H3K9me3 profiles (Figure S2D), suggesting that there are more cell-type-specific H3K27me3 features than H3K9me3 features.

Differential peak analysis identified subclass-specific H3K27me3 and H3K9me3 features that are globally distinguishing, from which we identified many more subclass-specific H3K27me3 peaks (289,341 features) than H3K9me3 peaks (89,025 features) (Table S4). We selected several cortical GABAergic neuronal subclasses for additional in-depth analysis due to their well-defined transcriptomic signatures, well-characterized developmental processes, and extensive data coverage across epigenomic modalities. We found 15,741 subclass-enriched H3K27me3 features (Figure 5C) and 11,354 subclass-depleted H3K27me3 features (Figure 5D), substantially more than subclass-enriched H3K9me3 features (1,073 regions) (Figure S6A) and subclass-depleted H3K9me3 features (763 regions) (Figure S6B). More interestingly, we found that subclass-specific H3K27me3 features frequently encompass genomic loci encoding transcriptional regulators and signaling molecules, suggesting that Polycomb-mediated gene repression plays important regulatory roles in shaping lineage-specific transcriptomes (Figures 5C, D).

We further examined various cell-type-specific H3K27me3 regions for their epigenomic landscapes and their target gene expression patterns (Figure S6C). *Lhx6*, encoding the transcription factor LIM homeobox 6 that serves as a key lineage-defining transcription factor for the MGE-derived GABAergic interneurons, is specifically expressed in neuronal cells of class 07 CTX-MGE GABA, such as subclasses 050 Lamp5 Lhx6 Gaba, 052 Pvalb Gaba, and 053 Sst Gaba. We found that the Chr-A state and its defining epigenomic marks (chromatin accessibility, H3K27ac, and H3K4me1) are specifically enriched at the *Lhx6* genomic locus. On the contrary, the CGE-derived GABAergic interneuron subclasses such as 046 Vip Gaba, 047 Sncg Gaba and 049 Lamp5 Gaba showed specific H3K27me3 deposition at the *Lhx6* genomic locus, which marked the local chromatin state as Hc-P, suggesting Polycomb-mediated repression of *Lhx6* transcription in CGE-Gaba interneurons (Figure S6C, left).

Genetically downstream of Lhx6, Sox6 is a key transcription factor in regulating the development, migration, and function of Pvalb and Sst interneurons^90,91^. Among CTX-CGE and CTX-MGE GABA neuronal subclasses, we found specific depletion of H3K27me3 marks on the *Sox6* genomic locus in neuronal subclasses 052 Pvalb Gaba and 053 Sst Gaba, as well as active chromatin signatures. Although subclass 050 Lamp5 Lhx6 Gaba is also derived from the Lhx6-marked MGE lineage, the epigenomic pattern on its *Sox6* locus showed strong H3K27me3 deposition and reduced active chromatin signatures, which makes it clearly distinct from other CTX-MGE GABA neurons, suggesting Polycomb-mediated repression in specifying different neuronal cell subclasses from the same neuronal cell class (Figure S6C, middle).

We also identified differential H3K27me3 coverage at a genomic locus located within the 5’ portion of the *Fgf12* gene body, which encodes fibroblast growth factor 12 (also known as fibroblast growth factor homologous factor 1), a key regulator of voltage-gated sodium channels involved in neuronal action potential initiation and propagation^92–94^. Interestingly, while the downstream half of the *Fgf12* genomic locus showed ubiquitous active chromatin signatures, the upstream half that encodes the long isoforms of *Fgf12* showed the strongest H3K27me3 signals in 049 Lamp5 Gaba and 050 Lamp5 Lhx6 Gaba subclasses and weakest to no signal in 052 Pvalb Gaba and 053 Sst Gaba subclasses. This pattern is negatively correlated with open chromatin, H3K27ac, and H3K4me1 signals as well as RNA-seq reads that were mapped to this region, suggesting elevated expression of *Fgf12* long isoforms in Pvalb and Sst GABAergic interneurons (Figure S6C, right). This expression pattern can be validated in our previous independent study, in which we performed single-cell multiomic analysis of the primary motor cortex across various mammalian species^11^. Comparing single-cell gene expression profiles of cortical GABAergic neurons from mouse, marmoset, macaque, and human, we found that the preferential RNA signals mapped to the *Fgf12* 5’ region in all four examined species, suggesting an evolutionarily conserved function of *Fgf12* long isoforms in Pvalb and Sst interneurons (Figure S6D).

Although we found very few H3K9me3 features that are globally distinguishing at the subclass resolution, we identified a substantial amount of H3K9me3 that is specifically enriched at the class resolution, suggesting cell-type demarcation by this histone mark at a higher taxonomic level. We identified prominent H3K9me3 across various large genomic regions with cell-type-specific enrichment patterns, including those that cover: large olfactory receptor (Olfr) and vomeronasal receptor type-1 (Vmnr1) gene clusters in class 01 IT-ET Glut neurons, various developmental regulators of the embryonic retina and forebrain such as *Irx5*, *Irx6* and *Six3* in class 06 CTX-CGE GABA neurons, multiple hepatic metabolic genes of the cytochrome P450 family in class 07 CTX-MGE GABA neurons, and neuronal developmental regulators such as *Bcl11b*, *Dlx1*, *Dlx2* and *Tfap2c* in class 31 OPC-Oligo cells (Figure 5E, left). These observations are consistent with the known role of H3K9me3 in silencing lineage-inappropriate genes through constitutive heterochromatin formation^88,95,96^. On the other hand, we found class-specific H3K9me3-depleted regions frequently cover genes that are specifically expressed in that lineage, such as *Bcl11b*, *Satb2*, *Tbr1* that are involved in forebrain neuron fate commitment in class 01 IT-ET Glut^67,68,97–99^; *Emx2*, *Neurod1*, *Prox1* and *Tmem108* that are involved in dentate gyrus development in class 04 DG-IMN Glut^67,69,100–103^; various endothelial development related genes in class 033 Vascular cells; and various immune-related factors in class 34 Immune cells (Figure 5E, right). Taken together, our analyses highlight a central role for repressive chromatin in refining and stabilizing brain cell identities.

### Sex-dependent histone modification features and gene expression in brain cell types

We investigated the sex differences in the deposition of multiple histone marks and in gene expression patterns across brain cell subclasses (Table S6). We excluded sex chromosomes from our differential histone modification peak analysis, as they differ in copy number between males and females and are subject to chromosome-wide processes such as dosage compensation, X-chromosome inactivation, and extensive constitutive heterochromatin on the Y chromosome. Across most brain cell subclasses, we identified fewer than 100 differential peaks for any given histone modification on autosomes (Figure S7A, left). This suggests that the autosomal epigenomic landscapes for the four profiled histone marks are broadly similar between males and females. A subset of subclasses showed more than 100 differential peaks for at least one histone modification, including 006 L4/5 IT CTX Glut, 007 L2/3 IT CTX Glut, 008 L2/3 IT ENT Glut, 014 LA-BLA-BMA-PA Glut, 022 L5 ET CTX Glut, 061 STR D1 Gaba, 062 STR D2 Gaba, 063 STR D1 Sema5a Gaba, 064 STR-PAL Chst9 Gaba and 318 Astro-NT NN (Figure S7A, left). We next performed differential gene expression analysis using the snRNA-seq profiles of the Paired-Tag dataset (Figure S7A, right). As expected, X-linked genes such as *Xist* and *Kdm6a* were consistently detected as the top sex-biased transcripts in nearly all subclasses (Figures S7B, C). Beyond these, most subclasses contained relatively few sex-differential genes and the observed effect sizes were generally modest, with log2 fold changes smaller than 0.5. Nevertheless, we found various genes with sex-biased expression patterns as well as active or repressive histone marks that corroborate the gene expression changes, such as *Kcnc2* in 006 L4/5 IT CTX Glut, *St6galnac3* in 008 L2/3 IT ENT Glut, *Sptbn2* in 014 LA-BLA-BMA-PA Glut, and *Adarb2* in 063 STR D1 Sema5a Gaba (Figures S7D–G). Taken together, these analyses uncover small yet detectable sex differences in the transcriptomes and epigenomes of various brain cell types.

### Transposable elements contribute to cell-type-specific regulatory programs

Transposable elements (TEs) make up a large fraction of the mammalian genome (37.5%) in the mouse genome^104–107^. While most TE regions are epigenetically silenced through mechanisms such as H3K9me3-mediated heterochromatin formation, certain TEs are being increasingly recognized as important regulators of gene expression and genome organization in various developmental and disease processes^108^. Consistent with these notions, we found Heterochromatin-H3K9me3 (Hc-H) to be the most enriched chromatin state on long interspersed nuclear elements (LINEs) and long terminal repeats (LTRs) regions, suggesting a major role of H3K9me3 in silencing these TEs (Figure 3B).

**Figure 6.**
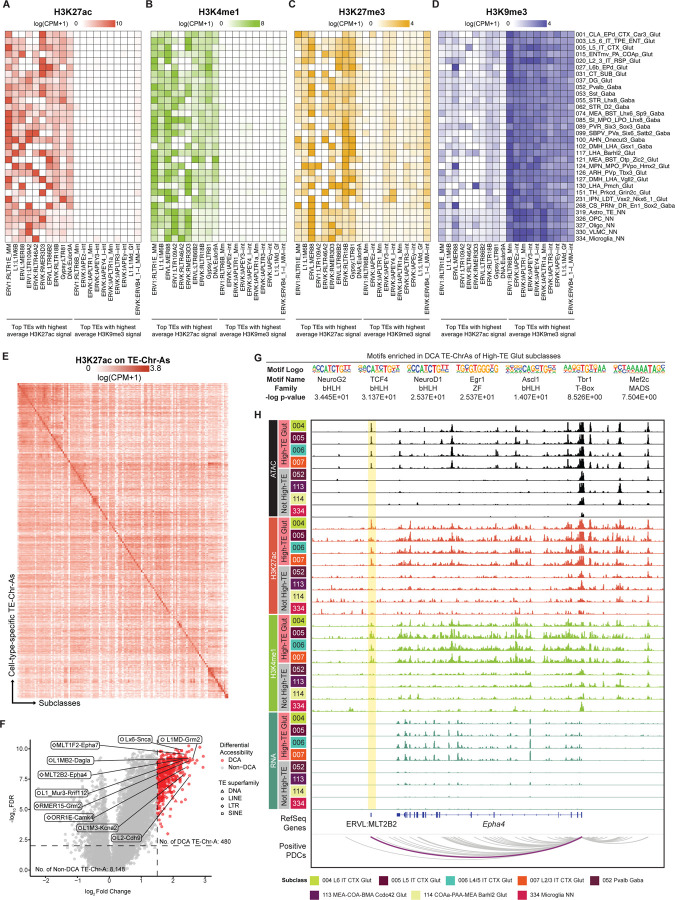
Transposable elements as potential gene regulatory elements. A–D. Heatmaps showing average H3K27ac (A), H3K4me1 (B), H3K27me3 (C), and H3K9me3 (D) signals across selected brain cell subclasses at the same set of transposable elements (TEs). TEs are grouped by those exhibiting the highest average H3K27ac or H3K9me3 signal across subclasses. E. Heatmap showing normalized H3K27ac signal at cell-type-specific TE-Chr-As across brain cell subclasses. F. Volcano plot showing differential chromatin accessible (DCA) TE-Chr-As in High-TE Glut subclasses compared with other subclasses. The top ten DCA TE-Chr-As correlated with synaptic-related genes are shown. G. Motifs enriched in DCA TE-Chr-As of High-TE Glut subclasses. Statistical significance was assessed using Fisher’s exact test. H. Genome browser tracks showing ATAC, H3K27ac, H3K4me1, and RNA signals for selected High-TE Glut subclasses (004 L6 IT CTX Glut, 005 L5 IT CTX Glut, 006 L4/5 IT CTX Glut, and 007 L2/3 IT CTX Glut) and representative subclasses that are not High-TE (052 Pvalb Gaba, 113 MEA-COA-BMA Ccdc42 Glut, 114 COAa-PAA-MEA Barhl2 Glut, and 334 Microglia NN), at the *Epha4* locus. An ERVL:MLT2B2 TE-Chr-A is highlighted, with specific ATAC, H3K27ac, and H3K4me1 signals in High-TE Glut subclasses, and a positive proximal-distal connection (PDC) to the *Epha4* transcriptional start site.

We have also previously reported a strong enrichment of TEs in mouse brain cCREs, especially in certain subclasses of excitatory neurons^17^. Using cell-type-resolved epigenomic landscapes of the mouse brain, we first examined H3K27ac and H3K9me3 signals across genomic regions of each TE subfamily as proxies of their overall activation and repression states, and evaluated their associated H3K4me1 and H3K27me3 levels. This analysis identified two distinct modules of TE subfamilies that feature the highest average H3K27ac (active) and H3K9me3 (silenced) signals among brain cell subclasses, respectively, suggesting that different subfamilies of TEs are subjected to distinct modes of regulation mediated by histone modifications (Figures 6A–D). Specifically, TE subfamilies in the H3K27ac-enriched module also exhibited elevated H3K4me1 signals (Figure 6B), consistent with features of enhancer-like, active chromatin states. In contrast, top TE subfamilies with the highest H3K9me3 signal showed uniformly low H3K27ac and H3K4me1 levels (Figures 6A, B), suggesting a H3K9me3-mediated constitutive heterochromatin state. Notably, TE subfamilies in the H3K27ac-enriched module exhibited higher overall H3K27me3 levels than those in the H3K9me3-silenced module; however, within the H3K27ac-enriched module, H3K27me3 signals were selectively reduced in the cell types where these TEs displayed high H3K27ac levels, resulting in an inverse relationship between H3K27me3 and H3K27ac across cell types (Figure 6C). Together, these data suggest that H3K27me3 confers facultative repression on TE subfamilies with enhancer-like chromatin signatures, enabling cell-type-specific deployment of TE-derived regulatory activity.

Previously, we have identified cCREs in the mouse brain that overlap with TEs (TE-cCREs)^17^. To further refine their regulatory heterogeneity, we leveraged the cell-type-resolved epigenomic landscapes of the mouse brain to characterize the chromatin states of TE-cCREs across brain cell subclasses, which classified TE-cCREs into two major chromatin state categories, TE-Chr-A and TE-Chr-O. Compared to TE-Chr-O elements, TE-Chr-As exhibited significantly higher levels of H3K27ac and H3K4me1 across brain cell types, suggesting stronger regulatory activity associated with the TE-Chr-As (Figures S7H, I). We also defined cell-type-specific TE-Chr-A elements for each brain cell subclass by their differential chromatin accessibility (Figure 4F, Table S7), and these TE-Chr-As also displayed pronounced cell-type-specific H3K27ac patterns (Figure 6E), suggesting that a subset of TE-derived regulatory elements is selectively deployed in a highly cell-type-dependent manner in the mouse brain.

We have also previously identified 22 subclasses of glutamatergic neurons across the mouse brain, the majority of which are cortical, that show a high fraction of cCREs overlapping with TEs; we refer to these as High-TE Glut subclasses (Figure S7J)^17^. Here, we further identified a higher fraction of Chr-As overlapping with TEs in High-TE Glut subclasses compared to the rest of the brain cell subclasses (not High-TE), further suggesting the role of identified TEs as gene regulatory elements (Figure S7K). Differential chromatin accessibility (DCA) analysis further identified 480 TE-Chr-As that are significantly enriched in High-TE Glut subclasses compared with not HighTE subclasses (Figure 6F). Within these DCA TE-Chr-As, TEs from the LINE superfamily (FDR = 9.06×10^−11^) and the L1 subfamily (FDR = 4.39×10^−9^) were preferentially enriched, consistent with our previous report that TEs from the L1 subfamily/LINE superfamily are preferentially enriched in DCA TE-cCREs in High-TE Glut subclasses^17^.

We further performed motif analysis on DCA Chr-As of High-TE Glut subclasses, and identified enrichment of many TF motifs with implications in neuronal development and function, such as NeuroG2, TCF4, NeuroD1, Egr1, Ascl1, Tbr1 and Mef2C (Figure 6G)^66,69,109–114^. In addition, we identified 81 DCA TE-Chr-As that showed strong positive proximal-distal connections to synaptic function-related genes, such as one MLT2B2 element found downstream of the *Epha4* genomic locus, suggesting its contribution to the preferential expression pattern of *Epha4* in High-TE Glut neuronal subclasses (Figure 6H).

Taken together, these data suggest that certain TEs are actively involved in gene regulation as potential enhancer elements that contribute to cell-type-specific gene expression patterns in the mouse brain.

### Predictive modeling of brain gene regulation from DNA sequence

**Figure 7.**
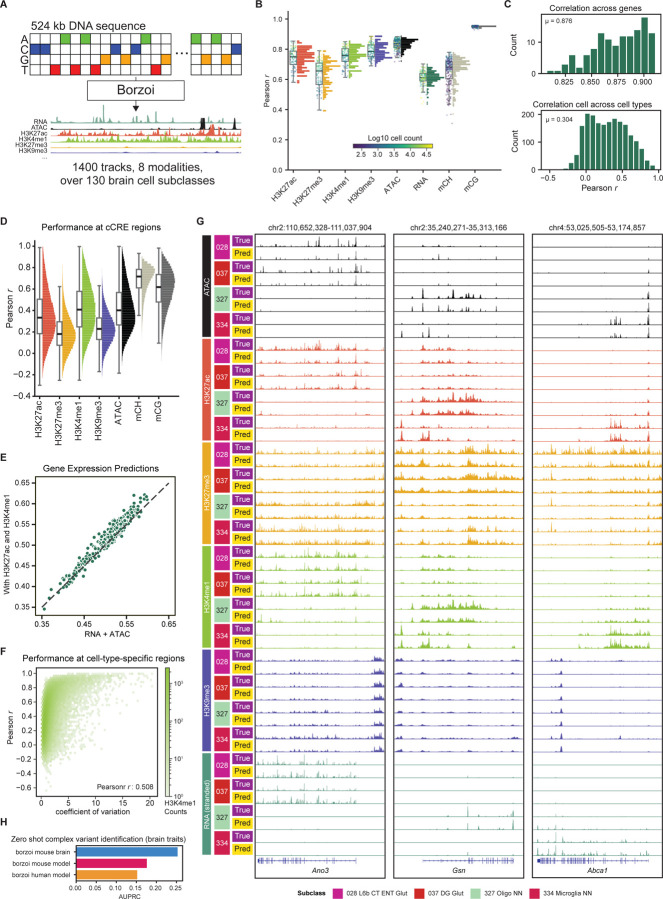
Deep learning prediction of brain epigenomic features and gene expression from DNA sequences. A. Schematic of the mouse brain Borzoi model, which takes as input 524 kilobases of DNA sequence and predicts 1400 tracks composed of 8 modalities in over 130 brain cell subclasses. Tracks are predicted as the average over every 32 base pairs. B. Model performance in an unseen test set for each modality was measured by the Pearson correlation between predicted values and measured values for each subclass. Boxplots show mean, inner quartile range and data extent of Pearson correlation across subclasses. Histograms show the distribution of correlation values, with point clouds highlighting each individual subclass. Each point is colored by the number of cells measured in each subclass. C. Top, histogram of the Pearson correlation between predicted expression and measured expression across all genes in the test set for each subclass. Bottom, histogram shows the Pearson correlation between the predicted expression pattern across cells and the measured expression pattern across cells for each gene in the unseen test set. D. Model performance in the unseen test set predicting patterns of epigenomes across cell types at mouse brain cCRE regions^17^. Boxplots show mean, inner quartile range, and data extent across cCRES with histograms showing the distribution. E. A comparison of gene expression prediction with ATAC alone or when adding H3K27ac and H3K4me1 when training from scratch. Points show the correlation between predicted and measured tracks in an unseen test set, with the x position showing the value for a model trained with ATAC alone, and the y position showing the value for a model trained with active histone modifications as well. F. Model performance across cCREs with different levels of variability. The Pearson R measures the model performance predicting patterns of H3K4me1 across cells, while the coefficient of variation measures the specificity of measured H3K4me1 across subclasses. G. Genome browser tracks showing predicted and measured ATAC, H3K27ac, H3K27me3, H3K4me1, H3K9me3, and RNA signals across representative brain cell subclasses (028 L6b CT ENT Glut, 037 DG Glut, 327 Oligo NN, and 334 Microglia NN) at selected cell-type specific genes (*Ano3*, *Gsn*, and *Abca1*) in the unseen test set. H. The model’s performance without tuning (zero-shot) on all neurological trait variants in the TraitGym benchmark compared to Borzoi, both the mouse model which served as the basis of the fine-tuned mouse brain Borzoi, and the human model.

Finally, we leveraged this multi-modal atlas to train sequence-based deep learning models that predict cell-type-specific epigenomic features and gene expression directly from DNA sequence. Sequence-to-function deep learning models present a major frontier in interpreting DNA function, with state-of-the-art performance in predicting regulatory function^115,116^, interpreting both coding and non-coding variation^117–119^, and designing functional DNA sequences *de novo*^120,121^. To leverage these capabilities, we fine-tuned the Borzoi model using the cell-type-resolved epigenomic maps generated by Paired-Tag together with other relevant BICCN data, comprising approximately 1,400 tracks spanning more than 130 subclasses, seven epigenomic modalities, and stranded gene expression. We preserved the original Borzoi architecture, which predicts epigenome profiles and RNA coverage tracks at 32 bp resolution using a combined Unet/Transformer architecture to integrate regulatory information across 524kb of input DNA sequence (Figure 7A).

Our model demonstrates strong performance on held-out test data, with predictions achieving an average correlation of 0.74 for H3K27ac, 0.63 for H3K27me3, 0.75 for H3K4me1, 0.78 for H3K9me3, 0.83 for ATAC, 0.60 for RNA, 0.65 for mCH and 0.94 for mCG between predicted and measured signals (Figure 7B). Prediction accuracy for a given cell type showed a strong correspondence to cell number (Figure 7B, Figure S8A), and an even stronger dependence on bigwig file size, which serves as an effective proxy for genome coverage. Reduced coverage increases quantification uncertainty and technical artifacts, as measured by the proportion of predicted signal overlapping with the ENCODE blacklist regions^122^, and the maximum RPKM for a prediction track (Figures S8B, C).

While RNA coverage was more challenging to predict than other epigenomic modalities, our model accurately predicted the relative expression of various genes within individual cell types, with an average Pearson correlation of 0.876 (Figure 7C). Predicting gene expression differences across cell types proved more difficult, with an average correlation per gene of 0.3 (Figure 7C). This behavior likely reflects a bias in model training to attend to highly expressed genes, which incur larger loss penalties, given that we found the model’s ability to predict expression differences across cells is highly correlated with the expression of a gene in its most highly expressed cell type (Figure S8E).

To assess our model’s ability to predict cell-type-specific regulatory elements, we measured the correlation between our model’s predicted epigenomes and the measured epigenomes at regions of our previously defined brain cCREs (Figure 7D)^17^. Our model demonstrated exceptional performance in predicting DNA methylation differences across cell types, potentially reflecting better sampling stability or cell-type-specific differences in methylation rate.

While predictions for active histone modifications were generally robust, our model’s performance was reduced for predicting differences in repressive histone marks, likely due to lower biological variability and weaker signal intensity at these regions (Figure S8F). For active histone marks, we found that our model’s ability to predict epigenome patterns at peaks across cell types was highly correlated with the variability of those regions across cell types, as measured by the coefficient of variation (Figure 7E, S8D). While incorporating multiple modalities yielded only limited additional gains when fine-tuning Borzoi, models trained from scratch using active histone marks (H3K27ac and H3K4me1) outperformed those trained on chromatin accessibility alone in predicting RNA expression (Figure 7F). To demonstrate the interpretability of our model’s predictions, we visualized predicted epigenomic and RNA tracks at representative gene loci (Figure 7G). Among these genes, *Ano3* encodes a calcium-activated chloride channel and phospholipid scramblase highly expressed in glutamatergic neurons and certain GABAergic neurons; *Gsn* encodes an actin-binding protein that is important for oligodendrocyte differentiation and maturation; and *Abca1* encodes a cholesterol efflux regulator that is preferentially expressed in non-neurons, with the highest expression in microglia. For each locus, the model-predicted RNA coverage and epigenomic tracks closely recapitulate measured signals, capturing cell-type-specific patterns of both active and repressive regulatory features (Figure 7G).

We further evaluated our model’s ability to identify complex causal variants using the TraitGym benchmark^117^, which consists of a set of fine-mapped, high-probability causal variants, along with nine other variants with similar properties (e.g., minor allele fraction). Causal variants are identified with no further training (zero-shot), by assuming the distance between a model’s predictions for a causal variant and its reference sequence should be greater than the distance between control variants and their reference sequence. When restricted to variants for neurological traits, our model achieved an area under the precision-recall curve of 0.25, a marked increase compared to the Borzoi with no additional tuning (0.17 and 0.14 for mouse and human sequence Borzoi models, respectively). In contrast, trained on brain-specific data, our model showed a decreased ability to detect causal variants overall, likely reflecting the small contribution of neurological traits within the full benchmark (Figure S8G). To facilitate broad access to these models, we provide an interactive online predictor that predicts subclass-specific epigenomes and transcriptomes for arbitrary DNA sequences at https://seqnn.org.

## DISCUSSION

In this study, we present a comprehensive single-cell, multi-modal epigenomic atlas of the adult mouse brain that integrates transcriptome with multiple layers of chromatin regulation. By jointly profiling gene expression and four major histone modifications and anchoring these data to existing brain maps of chromatin accessibility, DNA methylation, and 3D genome organization^6,16,17^, we establish a unified, cell-type-resolved framework for interpreting the regulatory genome of a complex mammalian tissue.

A central outcome of this work is the substantial expansion of functional genome annotation in the adult mouse brain. Whereas chromatin accessibility profiling has delineated large catalogs of cCREs, these approaches alone provide limited insight into regulatory state or mechanism. By integrating activating and repressive histone modifications, we functionally annotate approximately 81% of the brain genome and distinguish active, primed, and repressed chromatin states across diverse brain cell types. This expanded annotation suggests that cell-type identity is encoded not only through selective activation of regulatory elements, but also through widespread deployment and resolution of repressive chromatin programs.

Our analyses also clarify the functional distinction between chromatin accessibility and regulatory activity. Regions marked by active chromatin states, defined by the combinatorial presence of accessibility, H3K27ac, and H3K4me1, exhibit greater evolutionary conservation, stronger enrichment for experimentally validated brain enhancers, and higher cell-type specificity than accessible regions lacking these marks. In contrast, accessible regions lacking active histone marks are enriched for architectural features, including CTCF-associated motifs, suggesting roles in genome organization rather than in transcriptional activation. These findings highlight the importance of multiomic integration of epigenomic landscapes for accurate identification of functional enhancers and have broad implications for developing cell-type-specific delivery tools, dissecting gene-regulatory mechanisms underlying brain development and function, and interpreting non-coding regulatory variants in neurological and psychiatric disease. The approximately half million active chromatin regions identified here also constitute a rich set of candidate enhancers that can be prioritized for high-throughput validation of regulatory activity and cell-type specificity.

Beyond activation, our atlas reveals distinct and complementary roles for repressive chromatin programs in shaping brain cell identity, particularly those marked by H3K27me3 and H3K9me3. Polycomb-associated H3K27me3 exhibits pronounced cell-type specificity and preferentially targets transcriptional factors and signaling genes, underscoring its role in refining lineage-specific gene programs. In contrast, H3K9me3 demarcates broader lineage boundaries by silencing gene programs that are incompatible with a given cell identity. Together, these repressive mechanisms act in concert with active chromatin states to stabilize transcriptional programs in the adult brain.

The integrative framework further enables analysis of chromatin state dynamics across related cell states. Reconstruction of chromatin transitions during oligodendrocyte differentiation reveals coordinated remodeling of activating and repressive states at key lineage genes, closely paralleling transcriptional changes. These results illustrate how chromatin state transitions encode developmental progression and suggest that similar principles may underlie plasticity and state transitions in other neural lineages.

Our study also provides a refined view of TEs in brain gene regulation. While most TEs are embedded in H3K9me3-marked heterochromatin, we identified a subset of TE subfamilies that acquire enhancer-like chromatin signatures in a cell-type-specific manner. We further identified TEs with active marks that are cell-type-specific, particularly in certain glutamatergic neuronal subclasses (High-TE Gluts), and are enriched for transcription factor binding motifs relevant to neural development and function. These findings support an emerging view that TEs are not merely genomic parasites but can be selectively co-opted as gene-regulatory elements in specific cellular contexts, particularly in the mammalian brain. Our deep learning model captures a comprehensive, cell-type-resolved regulatory grammar across more than one hundred subclasses and seven epigenomic modalities, enabling prediction of gene expression and generalizing across species to support improved identification of genetic drivers of neurological traits.

Finally, by leveraging the scale and diversity of this atlas, we train sequence-based deep learning models that predict cell-type-specific epigenomic features and gene expression directly from DNA sequence. These models capture regulatory grammar beyond chromatin accessibility alone and provide a predictive framework for interpreting non-coding sequences in the context of defined brain cell types. Although predicting gene-expression differences across cell types remains challenging, this work demonstrates the value of multimodal epigenomic data for advancing sequence-to-function modeling in complex tissues.

Despite its scope, this study has limitations. First, although we sampled nine major brain regions, the atlas does not encompass the entire mouse brain; structures such as the cerebellum, olfactory bulb, and additional midbrain regions remain to be characterized. Second, our analyses focus on the adult brain (8 weeks) and therefore do not capture chromatin dynamics across the lifespan, which are critical for understanding cell fate specification, maturation, and brain aging. Third, although we profiled multiple major histone modifications, other epigenetic features with regulatory roles, such as additional histone marks, transcription factor occupancy, and chromatin-associated proteins, were not included. Notably, the modular design of Paired-Tag enables extension of this framework to additional epigenetic modalities in future studies.

In summary, this work establishes a comprehensive, cell-type-resolved functional annotation of the regulatory genome in the adult mouse brain. By integrating active and repressive chromatin states with transcriptome and predictive modeling, we provide a general framework for understanding gene regulation in complex tissues and a foundational resource for interpreting non-coding genetic variation in the mammalian brain.

## RESOURCE AVAILABILITY

### Lead contact

Requests for further information and resources should be directed to and will be fulfilled by the lead contact, Bing Ren (bren@nygenome.org).

### Materials availability

This study did not generate new unique reagents.

### Data and code availability

All software, sequencing data from this study, and the sources of publicly available data used in this study are listed in the key resources table. Raw data have been deposited to the NeMO Archive (RRID: SCR_016152) with the following collection IDs (nemo:col-wgpj7cd; nemo:col-xt1ksye, landing page: https://assets.nemoarchive.org/grant/nemo:grn-f309ksd). Processed data are available through CATlas web portal (https://catlas.org/catlas/amb-pt, RRID: SCR_018690). Custom codes and scripts used for analysis are available at GitHub (https://github.com/beyondpie/amb_pairedtag).

## Experimental model and subjects details

### Experimental animals

Animal work described in this manuscript has been approved and conducted in accordance with the Institutional Animal Care and Use Committee (IACUC) protocols at the Salk Institute. C57BL/6J mice (000664, RRID: IMSR_JAX:000664) were purchased from the Jackson Laboratories at 6 weeks of age and maintained in the Salk animal facility on a 12-h dark/light cycle with controlled temperature (20–22 C) and humidity (30–70%), and with *ad libitum* access to food and water.

### Cell lines and cell culture

HeLa S3 (ATCC, CCL-2.2) cells were cultured in Dulbecco’s Modified Eagle Medium (ThermoFisher Scientific, 11995073) supplemented with 10% fetal bovine serum (Omega Scientific, FB-02) and 1% penicillin-streptomycin-glutamine (ThermoFisher Scientific, 10378016), at 37 C with 5% CO2. Cells were not authenticated or tested for mycoplasma.

## Method details

### Mouse tissue preparation and nuclei isolation

Brains of C57BL/6J mice were collected from 8-week-old mice and sectioned into 600 μm coronal sections along the anterior-posterior axis in ice-cold dissection medium [20 mM Sucrose, 28 mM D-Glucose (Dextrose), 0.42 mM NaHCO3, in HBSS]^17,138^. Specific brain regions were dissected according to the Allen Brain Reference Atlas^46^, and stored at −80°C, as previously described^16,17^. For nuclei isolation of each brain region, dissected brain tissues were pooled from 2–8 animals of the same sex to obtain enough nuclei for Paired-Tag experiment for each biological replica, and two biological replicas were performed for both male and female samples. Frozen brain tissue samples were first dounce-homogenized with pestle A x 5–10 times and pestle B × 15–20 times in ice-cold Douncing Buffer [0.25 M sucrose (Sigma, S7903), 25 mM KCl (Invitrogen, AM9640G), 5 mM MgCl2 (Invitrogen, AM9530G), 10 mM Tris-HCl pH 8.0 (Invitrogen, 15568025), 1 mM DTT (Sigma, 43816), 1× cOmplete Protease Inhibitor cocktail (Roche, 11873580001), 0.5 U μL^−1^ RNase OUT (Invitrogen, 10777–019) and 0.5 U μL^−1^ SUPERase Inhibitor (Invitrogen, AM2694)] supplemented with 0.1% Triton X-100 (Sigma, 93443). Homogenates were then filtered through 30-μm Cell-Trics filters (Sysmex, 04–004-2326) and spun down for 10 min at 1,000 × g and 4°C. The nuclei pellets were resuspended with an equal volume of ice-cold Douncing Buffer and spun down again. The washed nuclei pellets were permeabilized in ice-cold Nuclei Isolation Buffer (NIB) [5% bovine serum albumin (Sigma, A1595) in PBS (ThermoFisher Scientific, 14190250), 0.2% IGEPAL CA-630 (Sigma, I8896), 1× cOmplete Protease Inhibitor cocktail, 1 mM DTT, 0.5 U μL^−1^ RNase OUT, and 0.5 U μL^−1^ SUPERase Inhibitor] for 5 min. The nuclei were counted on a cell counter (RWD, C100) upon DAPI staining and subjected to Paired-Tag experiments immediately.

### Spike-in HeLa cell preparation

HeLa S3 cells from passage 1 to 25 were used as spike-in control cells in the Paired-Tag experiment. HeLa cells were dissociated with 0.25% Trypsin-EDTA (ThermoFisher Scientific, 25200056), washed with PBS, and incubated in ice-cold NIB for 5 min. The HeLa nuclei were counted on a cell counter upon DAPI staining and subjected to Paired-Tag experiments immediately.

### Paired-Tag experimental procedures

Paired-Tag experiments were performed as previously described^35^, with major modifications in barcode design to improve the throughput, as detailed below.

#### Oligo sequences and assembly of transposomes

All oligo sequences used can be found in Supplementary Table 1. Specifically, 48 newly designed barcoded DNA adaptor oligos were reconstituted with nuclease-free H2O (ThermoFisher Scientific, AM9930), mixed with a pMENTs oligo individually (final concentration of 50 μM) and annealed with the following program using a thermocycler: 95°C × 5 min, then slowly ramp down to 4°C at a speed of −0.1°C s^−1^. Then, annealed adaptor oligos were mixed with unloaded Protein A-Tn5 (0.7 mg mL^−1^) in a 1:6 volume ratio. The mixtures were briefly vortexed and spun down with a desktop centrifuge, incubated at room temperature for 30 min then at 4°C for an additional 10 min. The assembled transposome complexes (also referred to as barcoded pA-Tn5) were stored at −20°C for up to 6 months. For barcoded reverse transcription (RT) primers, 48 newly designed barcoded poly-dT RT primers and 48 newly designed barcoded random hexamer RT primers were reconstituted at 100 μM, then mixed and diluted with nuclease-free water correspondingly to make 48 barcoded RT primer mixes, with both poly-dT and random hexamer RT primers at a final concentration of 12.5 μM.

#### Antibody-pA-Tn5 incubation and targeted tagmentation

For each Paired-Tag experiment, usually 16–32 reactions were included, where each reaction profiled one sample and one histone modification. The following histone modification antibodies were used: anti-H3K27ac (Abcam, ab4729), anti-H3K27me3 (Abcam, ab195477), anti-H3K4me1 (Abcam, ab8895), anti-H3K9me3 (Abcam, ab8898).

For each Paired-Tag reaction, 2 μg antibody and 1 μL barcoded pA-Tn5 were diluted in 30 μL MED1 buffer [1% BSA, 300 mM NaCl, 20 mM HEPES pH 7.5 (Invitrogen, 15630106), 1× cOmplete Protease Inhibitor cocktail, 0.01% Digitonin (Sigma, 300410), 0.5 mM Spermidine (Sigma, 05292), 2 mM EDTA (ThermoFisher Scientific, 15575020), 0.01% IGEPAL CA-630, 1 mM DTT, 0.5 U μL^−1^ RNase OUT, and 0.5 U μL^−1^ SUPERase Inhibitor] and pre-incubated on a tube rotator for 1 h at room temperature. 500–800k nuclei from each sample were resuspended in 45 μL MED1 buffer, mixed with pre-incubated antibody-pA-Tn5-MED1 mix, and further incubated overnight on a tube rotator at 4°C. Within each Paired-Tag experiment, we usually spike in ~10% HeLa nuclei (50–80 k) to mouse brain nuclei in one or two reaction tubes to estimate the barcode collision rate of the entire experiment. After this incubation, the nuclei were spun down at 500 × g at 4°C for 10 min, washed twice with 75 μL MED2 buffer (2% BSA, 300 mM NaCl, 20 mM HEPES pH 7.5, 1× cOmplete Protease Inhibitor cocktail, 0.01% Digitonin, 0.5 mM Spermidine, 0.01% IGEPAL CA-630, 1 mM DTT, 0.5 U μL^−1^ RNase OUT, and 0.5 U μL^−1^ SUPERase Inhibitor), and resuspended in 75 μL MED2 buffer. The tagmentation reaction was activated by adding 3 μL 250 mM MgCl_2_ and was carried out at 550 rpm, 37°C for 1 h in a ThermoMixer (Eppendorf). Following tagmentation, 24.8 μL of 40.4 mM EDTA was added to quench the reaction. Nuclei were then spun down at 500 × g at 4°C for 10 min and proceeded to reverse transcription immediately.

#### Reverse transcription

For each Paired-Tag reaction, nuclei were resuspended in 24 μL RT mix [6 μL 5x RT buffer, 6.9375 μL nuclease-free H_2_O, 6 μL PBS, 1.5 μL 10 mM dNTPs (NEB, N0447S), 0.375 μL SUPERase Inhibitor, 0.1875 μL RNase OUT, 3 μL Maxima H minus reverse transcriptase (ThermoFisher Scientific, EP0752)], and 6 μL of corresponding barcoded RT primer mix was added to each reaction with gentle pipetting to mix. RT reaction was performed in a thermocycler using the following program: Step 1, 50°C × 10 min; Step 2, 8°C × 12 s, 15°C × 45 s, 20°C × 45 s, 30°C × 45 s, 42°C × 2 min, 50°C × 5 min, repeat for 3 total cycles; Step 3, 50°C × 10 min; Step 4, hold at 12°C. After RT reaction, the nuclei were pooled to a pre-chilled 1.5 mL Maxymum recovery tube (Axygen, MCT-150-L-C), and 5% Triton X-100 were added to the nuclei pool to a final concentration of 0.1%. Nuclei were then spun down at 500 × g at 4°C for 10 min and proceeded to combinatorial barcoding immediately.

#### Ligation-based combinatorial barcoding

Combinatorial barcodes were prepared as previously described^35^, and 5 μL annealed combinatorial barcode was distributed to each well of a DNA lobind 96-well plate (Eppendorf, 0030129504) using a Biomek i7 automated liquid handler (Beckman Coulter). Combinatorial barcoding oligo sequences can be found in Supplemental Table 1. In total, 4 × R02 barcoding plates (384 different R02 barcodes) and 4 × R03 barcoding plates (384 different R03 barcodes) were prepared and stored at −20°C. R02 and R03 barcoding plates were thawed and equilibrated to room temperature before use.

Post-RT nuclei were resuspended in 2 mL of 1x NEBuffer 3.1 (NEB, B6003S) and then transferred to the ligation mix [4500 μL H_2_O, 1000 μL 10x T4 Ligase Buffer (NEB, B0202S), 200 μL 10x NEBuffer 3.1, 100 μL Recombinant Albumin (NEB, B9200S), T4 DNA Ligase (NEB, M0202L)]. Each 20 μL of ligation mix with nuclei was distributed to R02 barcoding plates using a multichannel pipette and incubated at 300 rpm for 30 min at 37°C in a ThermoMixer. Next, 5 μL of R02 blocking solution (prepared with 528 μL 100 μM R02 Blocking oligo, 500 μL 10x T4 Ligase Buffer, 972 μL H_2_O) was dispensed to each well using a multichannel pipette and the reaction was further incubated at 300 rpm for 30 min at 37°C in a ThermoMixer. The nuclei were then pooled and spun down at 500 × g at 4°C for 10 min. The next round of ligation-based combinatorial barcoding was performed using R03 barcoding plates similarly as the first round, except that after 30 min of the ligation reaction, 5 μL of R03 terminating solution (prepared with 528 μL 100 μM R03 Termination oligo, 1000 μL 500mM EDTA pH 8.0, 472 μL H_2_O) was added to each well to quench the ligation reaction. The nuclei were then collected by centrifugation at 500 × g, 4°C for 10 min. Post-barcoding nuclei were washed once in 1 mL PBS, resuspended in 1 mL PBS, and counted. Nuclei were aliquoted to sublibraries each containing 2,500 to 5,000 nuclei diluted to 35 μL in PBS. For each sublibrary, 5 μL 4M NaCl, 5 μL 10% SDS (Corning, 46–040-CI), and 5 μL Proteinase K (NEB, P8107S) were added, and the nuclei were lysed at 850 rpm, 55°C for 2 h to overnight in a ThermoMixer. The PK-digested sublibraries were cooled to room temperature, purified with 1x SPRIselect beads (Beckman Coulter, B23319), and eluted in 25 μL of H_2_O. The purified sublibraries were stored at −20°C, or proceeded for library preparation immediately.

#### Preamplification of barcoded DNA/cDNA

For each 25 μL purified sublibrary from the previous step, 3 μL 10x TdT Buffer and 1 μL 1 mM dCTP (NEB, N0446S) were added and the reaction was incubated at 95°C for 5 min, and then immediately chilled on ice for 5 min. Next, 1 μL Terminal Transferase (NEB, M0315L) was added, and the reaction mixture was incubated at 37°C for 30 min followed by 75°C for 20 min in a thermocycler. Subsequently, 30 μL Anchor Mix [14.4 μL H_2_O, 12 μL 5x KAPA HiFi Fidelity Buffer, 1.2 μL KAPA dNTPs Mix, 1.2 μL 10 μM Anchor-FokI-GH oligo, and 1.2 μL KAPA HiFi HotStart DNA Polymerase (Roche, KK2502)] was added to each reaction and the following thermocycling steps were performed in a thermocycler: Step 1, 98°C × 3 min; Step 2, 98°C × 15 s, 47°C × 1 min, 68°C × 2 min, 47°C × 1 min, 68°C × 2 min, repeat for a total of 16 cycles; Step 3, 72°C × 10 min; Step 4, hold at 12°C. Immediately afterwards, 40 μL Preamplification Mix (22 μL H_2_O, 8 μL 5x KAPA HiFi Fidelity Buffer, 4 μL 10 μM PA-F oligo, 4 μL 10 μM PA-R oligo, 1 μL KAPA dNTPs Mix, 1 μL KAPA HiFi HotStart DNA Polymerase) was added to each reaction and the following thermocycling steps were performed: Step 1, 98°C × 3 min; Step 2, 98°C × 20 s, 62°C × 20 s, 72°C × 2 min, repeat for a total of 10 cycles; Step 3, 72°C × 2 min; Step 4, hold at 12°C. Preamplified sublibraries were purified with double size selection (0.2x + 0.65x, 20 μL + 65 μL beads) using SPRIselect beads, and eluted in 40 μL of H_2_O. From purified sublibraries, 1 μL of eluted DNA was used in a Qubit 1x dsDNA High Sensitivity Assay (Invitrogen, Q33231) to measure DNA concentration. For the remaining purified sublibraries, 4.5 μL CutSmart Buffer (NEB, B6004S) was added to each reaction, from which 19 μL of the reaction mixture was transferred to a new tube (hereafter referred to as the RNA sublibrary tube). The original tube holding the remaining reaction is referred to as the DNA sublibrary tube from this point onward.

#### Restriction enzyme digestion and purification of DNA and RNA sublibraries

For each DNA sublibrary tube, 1 μL SbfI-HF (NEB, R3642L) and 1 μL FokI (NEB, R0109L) were added. For each RNA sublibrary tube, 1 μL NotI-HF (NEB, R3189L) was added. The restriction enzyme digestion reactions were incubated at 37°C for 2 h to overnight, and further purified using 1.25x SPRIselect beads (25 μL beads into each RNA sublibrary tube, 31.3 μL beads into each DNA sublibrary tube). Both post-digestion DNA and RNA sublibraries were eluted in 10 μL H_2_O.

#### DNA sublibrary preparation

For each 10 μL purified, post-digestion DNA sublibrary, 10 μL P5 adaptor ligation mix (5 μL H_2_O, 2 μL 10x T4 Ligase Buffer, 1.5 μL 10 μM P5 Adaptor mix, 1.5 μL T4 DNA ligase) was added and the ligation reaction was carried out using the following thermocycling program: 4°C × 10 min, 10°C × 10 min, 16°C × 15 min, 25°C × 1h, hold at 4°C. P5 Adaptor mix was prepared by: (1) mixing 25 μL 100 μM P5-FokI oligo with 25 μL 100 μM P5c-NNDC-FokI oligo; (2) mixing 25 μL 100 μM P5A-FokI oligo with 25 μL 100 μM P5Ac-NNDC-FokI oligo; (3) mixing 25 μL 100 μM P5C-FokI oligo with 25 μL 100 μM P5Cc-NNDC-FokI oligo; (4) mixing 25 μL 100 μM P5T-FokI oligo with 25 μL 100 μM P5Tc-NNDC-FokI oligo; (5) annealing oligo mixtures from (1)-(4) with the following program using a thermocycler: 95°C × 5 min, then slowly ramping down to 4°C at a speed of −0.1°C s^−1^; (6) mixing four annealed oligo mixtures in a 1:1:1:1 ratio; then diluting from 50 μM to 10 μM final concentration.

The ligation product was then purified by 1.25x (25 μL) SPRIselect beads purification and eluted in 20 μL H_2_O. Next, 25 μL NEBNext Ultra II Q5 Master Mix (NEB, M0544X), 2.5 μL 10 μM TruSeq i7 indexing primer, and 2.5 μL 10 μM TruSeq i5 indexing primer were added to the eluted DNA sublibraries. The indexing PCR reaction was conducted with the following thermocycling program: Step 1, 98°C × 3 min; Step 2, 98°C × 10 s, 63°C × 30 s, 72°C × 1 min, repeat for a total of 12 cycles; Step 3, 72°C × 1 min; Step 4, hold at 12°C. The indexing PCR reaction was purified using 0.7x (35 μL) SPRIselect beads and eluted in 20 μL H_2_O. The size distribution of the final DNA sublibrary was examined using a High Sensitivity D1000 screentape (Agilent, 5067–5584) on a TapeStation 4200 device (Agilent), and library yield was examined using qPCR. Sequencing was performed with an Illumina NextSeq 2000, NovaSeq 6000 or NovaSeq X sequencer using the following setting: Read 1 – 100 cycles, Index 1 – 8 cycles, Index 2 – 8 cycles, Read 2 – 100 cycles.

#### RNA sublibrary preparation

From each 10 μL post-digestion RNA sublibraries, 5 μL was transferred to a new tube for downstream library preparation. On ice, 10 μL Tagment DNA buffer (TD) and 5 μL Amplicon Tagment Mix (ATM) from the Nextera XT DNA Library Preparation Kit (Illumina, FC-131–1096) were added to 5 μL RNA sublibrary with gentle pipetting to mix, and the reaction mix was incubated at 55°C for 5 min. Immediately after the tagmentation reaction, 5 μL Neutralize Tagment Buffer (NT) was added to each reaction, and the reaction was incubated at room temperature for 5 min. Next, 5 μL H_2_O, 15 μL Nextera PCR Mastermix (NPM), 2.5 μL 10 μM TruSeq i7 indexing primer, and 2.5 μL 10 μM Nextera i5 indexing primer were added to the reaction, and the indexing PCR reaction was conducted with the following thermocycling program: Step 1, 72°C × 3 min; Step 2, 95°C × 30 sec; Step 3, 95°C × 10 s, 55°C × 30 s, 72°C × 30 s, repeat for a total of 14 cycles; Step 4, 72°C × 5 min; Step 5, hold at 12°C. The indexing PCR reaction was purified using 0.7x (35 μL) SPRIselect beads and eluted in 20 μL H_2_O. The size distribution of the final RNA sublibrary was examined using a High Sensitivity D1000 screentape on a TapeStation 4200 device and library yield was examined using qPCR. Sequencing was performed with an Illumina NextSeq 2000, NovaSeq 6000 or NovaSeq X sequencer using the following setting: Read 1 – 100 cycles, Index 1 – 8 cycles, Index 2 – 8 cycles, Read 2 – 100 cycles.

### Processing and alignment of sequencing reads

Cellular barcodes from the sequencing reads were first extracted by matching the linker sequences adjacent to the cellular barcodes, which were then mapped to the cellular barcodes reference using bowtie^131^. Reads with more than 1 nucleotide mismatch were discarded. The adapter sequences were trimmed from 3′ of DNA and RNA libraries, with Poly-dT and random hexamer sequences further trimmed from 3′ of RNA libraries. Cleaned reads were mapped to the mouse GRCm38/mm10 reference genome with STAR^130^ for RNA or bowtie2^132^ for DNA. Duplicated reads were removed based on the mapped position, cellular barcode, PCR index, and UMI. RNA alignment files were converted to a matrix with cells as columns and genes as rows. DNA alignment files were converted to a matrix with cells as columns and 5-kb genomic bins as rows. A detailed, step-by-step Paired-Tag data processing pipeline can be found at: https://github.com/zwang0715/Paired-Tag.

### Quality control and doublet removal

For each sublibrary, cellular barcodes with 500 – 30,000 DNA features and 200 – 30,000 RNA features were retained. The initial double rate in each sublibrary was estimated using the spike-in Hela S3 cells as described in^139^. Species-specific barcodes were defined as barcodes with at least 75% of RNA reads mapped to the genome of the corresponding species. We next applied a modified version of Scrublet implemented in SnapATAC2^47^ to remove potential doublets in each sublibrary, using the estimated initial doublet rates described above. Briefly, more than 8,000 differentially expressed genes identified from a previous adult whole mouse brain single-cell RNA-seq study were used as features^6^. Spectral embedding was performed across barcodes from all sublibraries using *snapatac2.tl.spectral* with the parameters *n_comps* as 50 and *weighted_by_sd* as “TRUE”. Doublet scores were then predicted for each barcode within individual sublibraries using *snapatac2.preprocessing.scrublet*. Because the predicted doublet probabilities were generally small, consistent with the relatively low doublet rates in the Paired-Tag experiments, applying the default Scrublet thresholds resulted in few cells being flagged as doublets. Therefore, we manually removed barcodes with the highest predicted doublet scores such that the proportion of removed cells in each sublibrary matched the corresponding estimated doublet rate. In addition to the quality control steps described above, additional low-quality cells were removed during downstream clustering and integration, as described in the corresponding Methods sections.

### Iterative cell clustering

We implemented a five-round iterative clustering strategy using single-nucleus RNA expression profiles from Paired-Tag^6^. Briefly, all 2.5 million single nucleus RNA-seq profiles from Paired-Tag were used for the first round of clustering (L1 level) using a standard clustering workflow. In the second round (L2 level), independent clustering was performed for each of the 17 L1-level clusters. In the third round (L3 level), independent clustering was carried out for each of the 182 L2-level clusters. In the fourth round (L4 level), independent clustering was performed for 972 of the 1,041 L3-level clusters. In the fifth round (L5 level), independent clustering was performed for 400 of the 3,339 L4-level clusters. In total, this hierarchical procedure identified 4,302 cell clusters. Detailed procedures are described below.

#### Feature selection.

For L1-level clustering, we used more than 8,000 differentially expressed genes defined in the previous adult mouse brain scRNA-seq study^6^. For subsequent clustering levels, highly variable genes were selected from this gene set using *scanpy.pp**.highly_variable_genes* in *scanpy*^125^, with the parameter *flavor* as “seurat_v3”, which emulates the *FindVariableFeatures* function with the parameter *method* as “vst” in Seurat^124,140^. Because cell clusters became progressively smaller with increasing clustering depth, we selected the top 3,000 genes for L2-level clustering and the top 2,000 genes for L3-, L4-, and L5-level clusterings.

#### Dimensionality reduction.

We applied *snapatac2.tl.spectral* from SnapATAC2 to project high-dimensional, sparse gene expression profiles into low-dimensional space. This approach performs spectral embedding of the normalized graph Laplacian defined by the cell-to-cell similarity matrix based on the cosine distance of highly variable genes’ expression. For L1-level clustering, the dimensionality of the embedding was set to 50, whereas a dimensionality of 30 was used for subsequent clustering levels. The parameter *weighted_by_sd* in the function was set to “TRUE”, as recommended.

#### Graph-based clustering.

We constructed k-nearest neighbor graphs using *snapatac2.pp.knn* from SnapATAC2 with the parameter *method* as “kdtree”. For L1- and L2-level clusterings, the parameter *n_neighbors* was set to 50, whereas a value of 30 was used for subsequent clustering levels. Clustering was then performed using *snapatac2.tl.leiden* with *objective_function* as “modularity”. Because the clustering resolution strongly influences the number of identified clusters, we evaluated a range of resolution values from 0.1 to 2 in increments of 0.1. Clustering performance at each resolution was assessed using the Silhouette coefficient as implemented in scikit-learn^136^, together with visual inspection of two-dimensional UMAP projections. UMAP embeddings were computed from the spectral embeddings using the umap package^137^ with parameters a as 1.8956, b as 0.8005, and init as “spectral”. For each clustering on different levels, the final resolution was selected based on a combination of Silhouette scores and UMAP visualization.

#### Histone modification-based clustering.

Single-cell histone modification profiles generated by Paired-Tag were processed using SnapATAC2 for dimensional reduction and clustering. Briefly, the genome was segmented into fixed-width bins, with a bin size of 5 kb for H3K27ac and H3K4me1, and 50 kb for H3K27me3 and H3K9me3, reflecting the distinct genomic distribution patterns of active and repressive histone marks. Blacklisted genomic regions were excluded, and only autosomal regions were retained for downstream analysis. For each histone modification, cell-by-bin count matrices were constructed and highly variable genomic bins were selected. Specifically, the top 250,000 most variable bins were retained for H3K27ac and H3K4me1, whereas the top 25,000 most variable bins were retained for H3K27me3 and H3K9me3. Dimensional reduction was then performed using spectral embedding as implemented in SnapATAC2. A k-nearest neighbor graph was constructed based on the spectral embedding, followed by Leiden clustering to identify cell clusters. For visualization, two-dimensional embeddings were generated using UMAP based on the same low-dimensional representation. To assess the concordance between histone mark-based embeddings and RNA-defined cell-type annotations, we computed normalized mutual information (NMI) and k-nearest neighbor (kNN) label transfer accuracy. NMI was calculated between Leiden cluster assignments and RNA-based cell-type labels using the *normalized_mutual_info_score function* in the *scikit-learn* package, with arithmetic mean normalization. kNN label transfer accuracy was computed by assigning each cell the majority RNA-based label of its k nearest neighbors in the SnapATAC2 spectral embedding space (k=15), excluding the cell itself. Only cells with valid labels in both modalities were included in the analysis.

### Transfer label-based annotation with scRNA-seq data

We performed the transfer label-based annotation for more than 4,000 Paired-Tag-derived cell clusters using a published adult whole mouse brain scRNA-seq dataset as reference^6^. The nine brain regions profiled by Paired-Tag were mapped to their corresponding anatomical regions in the scRNA-seq dataset as follows: prefrontal cortex (PFC) to ACA and PL-ILA-ORB regions in the scRNA-seq study, hippocampus anterior and posterior (HCa and HCp) to HIP, entorhinal cortex (ERC) to ENT, nucleus accumbens (NAC) to STRv,caudate putamen (CPU) to STRd, amygdala (AMY) to STR-sAMY and CTXsp, ventral tegmental area-substantia nigra (VTR_SnR) to MB-VTA_SN, and Hypothalamus (HYP) to HY. Only cells from both the mapped regions and generated using the 10x Genomics single cell 3’ gene expression v3 platform were retained from the scRNA-seq dataset, resulting in 656,346 cells used for downstream analysis while preserving all relevant cell clusters. Transfer label analysis was performed separately for neuronal and non-neuronal cells. Neuronal and non-neuronal populations were distinguished based on *Snap25* expression in L1-level Paired-Tag clusters.

For non-neuronal cells, we randomly sampled 30 cells in each of more than 1,300 L5-level clusters in the Paired-Tag dataset and 1,000 cells in each of L4-level clusters in the scRNA-seq data (including non-neuronal and immature-neural cells) to keep the cells from each dataset roughly balanced. More than 8,000 genes previously defined from differential expression analysis in the scRNA-seq study were used as variable features. We applied the *FindTransferAnchors* function in Seurat using canonical component analysis (CCA) to co-embed the two datasets into a joint low-dimensional space, followed by *TransferLabel* to annotate Paired-Tag cell with the supertype-level label from the scRNA-seq data. For each L5-level cluster, the most frequently assigned supertype label was selected as the cluster annotation. Co-embedding results were visualized using Uniform Manifold Approximation and Projection (UMAP), computed from the joint embedding generated by *FindIntegrationAnchors* as recommended in Seurat. Annotations were further validated by manual inspection of canonical marker gene expression.

For neuronal cells, a similar transfer label procedure was applied but performed separately within each brain region, reflecting the strong regional specificity of neuronal cell types. Within each region, cells from the reference scRNA-seq dataset with region-mismatched annotations were first ignored, and L5-level clusters (about 0.69% cells) from Paired-Tag dataset with small number of cells were flagged and excluded from our later analysis. To retain representative populations while balancing cell numbers, we down-sampled L5-level clusters from both Paired-Tag and reference scRNA-seq datasets, including immature neuronal cells. Variable features were then selected independently for each dataset using *FindVariableFeatures* in Seurat, retaining the top 3,000 genes per dataset. The intersection of these features was used for subsequent integration. L5-level clusters were annotated using subclass-level labels transferred from the scRNA-seq data. Clusters containing fewer than ten cells in each region were removed from further analysis. A small fraction (less than 0.01%) of Paired-Tag neuronal cells lacked corresponding co-embedded cells in the reference scRNA-seq dataset, likely reflecting differences in sample collection strategies between the two studies. These cells were instead annotated using transfer label results from a region-agnostic integration, following the same procedure applied to non-neuronal cells.

Additional manual quality control was performed for neuronal populations. For each brain region, major cell classes were projected onto the L1-level UMAP embedding of the Paired-Tag data. Cells (about 4.26% cells) deviating from the dominant cluster structure for their assigned class were flagged and excluded from downstream analyses. After transfer label-based annotations, we had 2,571,700 cells and 4247 clusters.

### Joint co-embedding of multiple single-cell modalities

To evaluate the integration of multiple single-cell modalities, we visualized their joint embeddings following Seurat-based integration. Because of the large scale of the datasets analyzed in this study, we applied a customized Python implementation of the Seurat integration workflow (https://github.com/scverse/SnapATAC2/tree/main/snapatac2contrib/snapatac2_contrib/integration)^124^.

We first confined all datasets to cells derived from the nine major brain regions described in the transfer-label analysis. After filtering, the datasets included 656,346 cells from scRNA-seq^6^, 63,792 cells from snmC-seq3^16^, 472,528 from snATAC-seq^17^, and 2,571,700 cells from Paired-Tag.

Next, gene expression levels were simulated for the snATAC-seq and snmC-seq3 datasets. For snATAC-seq, gene activity scores were computed from the number of fragments overlapping each gene body and its 2kb upstream region from the TSS, using the function *snapatac2.pp.make_gene_matrix* in SnapATAC2. For snmC-seq3 data, distinct strategies were used depending on cell type. In neuronal cells except granule neurons (DG Glut), gene expression is inversely correlated with the mCH fraction within the gene body. In non-neuronal cells (NN), immature neurons (IMN) and granule neurons, gene expression is inversely correlated with the mCG fraction in the gene body. Gene bodies were defined as regions spanning 2kb upstream of the TSS to 2kb downstream of the TES. After grouping cells according to these categories, the natural logarithm of the corresponding mCH or mCG fractions was z-score transformed, and the negative values were used as gene activity scores, following previously described methods^16^.

For data integration, the adult mouse brain scRNA-seq dataset was used as the reference, while snATAC-seq, snmC-seq3, and Paired-Tag datasets were treated as queries. Integration anchors were identified using canonical correlation analysis (CCA) with the function *integration.SeuratIntegration.find_anchor*, corresponding to *FindIntegrationAnchors* in Seurat. Only highly variable features shared between the reference and query datasets were used. To mitigate biases introduced by unequal cell numbers across subclasses, we performed downsampling at the cell subclass level for all datasets before integration.

Finally, the identified anchors were used to correct principal components for each query dataset using the function *integration.SeuratIntegration.integrate*, corresponding to *IntegrateData* in Seurat v4. Principal component analysis was performed independently for each modality using more than 1,500 highly variable genes shared across all datasets. The corrected principal components from all four modalities were then merged, and Harmony was applied to further reduce batch effects^126^. UMAP was performed on the Harmony-corrected embeddings to visualize cells from all modalities in a shared two-dimensional space.

### Identification of reproducible peaks for each histone modality

Peak calling was performed following the ENCODE pipeline separately for each sex, histone modification (four in total), and cell subclass. Sex-specific peaks were first identified and then merged to generate subclass-specific peak sets. These subclass-specific peaks were subsequently combined across cell subclasses to derive histone modification–specific peak sets. Detailed procedures are described below.

#### Sex-specific peak calling.

For each combination of sex, histone modification, and cell subclass, we first generated a pooled BAM file containing reads from all barcodes within that group. This pooled BAM was then split into four BAM files: two biological replicates and two shuffled pseudo-replicates generated by randomly and evenly splitting barcodes. Replicates with insufficient read depth were excluded from peak calling based on scatter plots of peak number versus read count across BAM files for different histone modifications. Minimum read thresholds were set to 10,000 reads for H3K27ac and 10^4.5^ (31,622) reads for the remaining histone modifications. Peak calling was performed separately for each retained bam files using MACS3^128^ with the following parameters: *--shift −100 --extsize 200 --nomodel --nolambda --keep-dup--all -g mm -q 0.05*. We compared peak calling results obtained with and without the *--nolambda* parameter. Visual inspection showed that inclusion of *--nolambda* retained the majority of biologically relevant peaks without compromising specificity, whereas exclusion of this parameter resulted in fewer detected peaks and loss of signal, likely due to the sparsity of DNA fragments in the dataset. Therefore, *--nolambda* was used in all downstream analyses. Given the distinct chromatin characteristics of different histone modifications, narrow peaks were called for H3K27ac by adding the *--call-summits* parameter, whereas broad peaks were called for the remaining histone modifications using *--broad --broad-cutoff 0.1*. Reproducible peaks were defined as those that satisfied both of the following criteria: (1) a MACS3 negative log10 q-value of at least 0.01, and (2) detection in all but at most one replicate, defined as overlap with at least one peak in the corresponding replicate peak sets.

#### Peak merging.

Peaks from different cell subclasses were merged using an iterative way implemented in ArchR^127^. This approach uses SPM scores, defined as normalized MACS peak scores or negative log10 q-values, to select representative peaks among overlapping regions, resulting in a non-overlapping and high-confidence peak set. For H3K27me3 and H3K9me3, peaks located on chromosome X were excluded. Peaks overlapping ENCODE blacklist regions were also removed. This merging strategy was first applied to generate unified peak sets for each cell subclass within each histone modification and was then applied again to generate a final unified peak set for each histone modification across all cell populations.

### Chromatin state annotation using ChromHMM

We applied ChromHMM^48^ to annotate chromatin states across the mouse autosomes for each cell subclass by integrating chromatin accessibility from single-nucleus ATAC-seq with histone modification profiles of H3K27ac, H3K27me3, H3K4me1, and H3K9me3 generated by Paired-Tag. Sex chromosomes were excluded from this analysis due to chromosomal-wide processes, such as X-inactivation, on sex chromosomes. Peaks from each modality were first used to generate binarized genomic tracks using the BinarizeBed utility in ChromHMM. Chromatin state models were then inferred using the LearnModel utility. To determine the optimal number of chromatin states, we evaluated a series of models with different state numbers. A 25-state model was selected as a “full” model, which captured all potential chromatin states with redundancy based on empirical observations. Models with 5 to 24 states were then compared with the full model using two complementary approaches, as described previously^27^. First, Pearson correlations were computed between emission probability vectors of each state in a query model and each state in the full model. For each state in the full model, the maximum correlation with any state in the query model was used to quantify how well that state was represented. The average of these maximum correlations across all full-model states was used as a summary metric, with a value of 1 for the full model. Query models with average correlations greater than 0.85 were considered acceptable, and models with average correlations greater than 0.99 were considered satisfactory. Second, k-means clustering was applied to group states within each query model and the full model using emission probabilities, with k equal to the number of states in the model. Clustering performance was quantified using the ratio of between-cluster sum of squares to total sum of squares. The full model served as a reference for maximal separation, and query models achieving at least 95% of the full model’s separation were considered acceptable. The minimal number of states (18) satisfying both criteria was selected as the optimal ChromHMM model.

To facilitate biological interpretation and downstream analyses, we collapsed the original 18-state ChromHMM model into a simplified 8-state chromatin annotation scheme with descriptive state names. State consolidation was guided by the emission probabilities of epigenomic marks, signal patterns observed in genome-wide coverage heatmaps, and the functional association of each state with gene activity. Specifically, Original states 3, 4, 5, 9, 12, and 13 were classified as active chromatin (Chr-A). These states were characterized by strong H3K27ac enrichment accompanied by co-present or flanking ATAC-seq and H3K4me1 signals, consistent with active regulatory elements. Although state 5 did not exhibit a prominent H3K27ac signal in the emission heatmap, inspection of the coverage heatmap revealed a diffuse yet robust H3K27ac signal across these regions, supporting its inclusion as active chromatin. State 9 displayed detectable H3K27me3 emission; however, this state also showed substantially higher ATAC, H3K27ac, and H3K4me1 signals compared with other states and was strongly associated with loci harboring highly expressed genes. Based on these combined features, state 9 was likewise classified as Chr-A. Original states 6 and 8 were classified as poised chromatin (Chr-Po) due to the co-occurrence of H3K4me1 and H3K27me3 signals, together with either chromatin accessibility or H3K27ac enrichment, a pattern consistent with poised regulatory elements. Original state 2 was classified as primed chromatin (Chr-Pr) based on the exclusive emission of H3K4me1, indicative of enhancer priming in the absence of overt activation. Original state 11 was classified as open chromatin (Chr-O) due to its exclusive ATAC-seq signal, reflecting accessible chromatin without accompanying histone modifications. Original states 7, 10, and 14 were grouped as repressed chromatin (Chr-R). These states were defined by strong H3K27me3 enrichment together with the presence of a single active chromatin-associated mark: H3K4me1 in state 7, ATAC-seq in state 10, and H3K27ac in state 14. This combination suggests a repressive chromatin environment with limited or contextdependent accessibility. Original states 15 and 16 were classified as heterochromatin–Polycomb (Hc-P) due to strong H3K27me3 emission in the absence of additional active chromatin marks. Although state 16 also exhibited H3K9me3 emission, it encompassed very few genomic regions and was therefore grouped with Polycomb-associated heterochromatin. Original state 17 was classified as heterochromatin–H3K9me3 (Hc-H), as it was uniquely defined by exclusive H3K9me3 enrichment. State 0 was designated as not determined (ND) because no epigenomic marks were detected. State 18 was also classified as ND due to the simultaneous detection of all assayed epigenomic marks, including ATAC-seq, H3K27ac, H3K27me3, H3K4me1, and H3K9me3, which we were not able to confidently assign to a single descriptive chromatin state category. Notably, this state covered less than 0.1% of the genome and was preferentially localized at highly expressed gene loci. We reasoned that this state represents a rare and negligible genomic fraction arising from the intrinsic open chromatin bias of Tn5-based single-cell genomic assays, and merged it into the ND chromatin state for accuracy. After consolidating the chromatin states, the corresponding emission matrix was recalculated by mapping original state assignments to the 8-state model and re-estimating emission probabilities using binarized signals from each modality.

### Variations of ChromHMM States across cell subclasses

To quantify conservation of ChromHMM states across cell subclasses, we calculated the proportion of overlapping base pairs between subclasses for each chromatin state. For a given ChromHMM state, cell subclass A was treated as the reference and subclass B as the comparison. For each genomic interval belonging to that state in subclass A, the most overlapping interval with the same state in subclass B was identified, and the number of overlapping base pairs was recorded, with a value of zero assigned if no overlap was detected. The conservation proportion between subclasses A and B was defined as the sum of overlapping base pairs divided by the total length of all intervals for that state in subclass A. State variation for subclass A was then quantified as the variance of conservation proportions across all other subclasses.

### Annotation of *cis-*regulatory elements (CREs) using ChromHMM states

For each cell subclass, *cis-*regulatory elements were annotated based on the ChromHMM state assigned to the peak summit, defined as the center of the CRE. The majority of CREs were annotated as Chr-O or Chr-A, followed by Chr-R and Chr-B. Only a small fraction of CREs were annotated as Hc-P or Hc-H, and no CREs were assigned to the Chr-P state.

### Analysis of differential regions using SnapATAC2

Differential region analysis was performed using *snapatac2.tl.diff_test* on peaks identified for each cell subclass and each DNA modality, including ATAC, H3K27ac, H3K4me1, H3K27me3, and H3K9me3. To identify cell subclass–specific differential regions, the *direction* parameter was set to “*positive*” for ATAC, H3K27ac, and H3K4me1, focusing on regions exhibiting significantly higher signal in the target subclass compared with all other cells; the *direction* parameter was set to “*both*” for H3K27me3 and H3K9me3 to characterize regions exhibiting significantly higher or lower signals in the target subclass/class compared with all other cells. The parameters *min_pct = 0.01* was used during differential testing. Differential regions were then defined using an adjusted p-value threshold of 0.05 and a minimum log fold change of 0.2 for each DNA modality. Differential regions were subsequently integrated with ChromHMM annotations. Using this framework, 470,176 cCREs were classified as distal subclass-specific Chr-As, defined as Chr-A overlapping subclass-specific differentially accessible CREs located more than 1 kb upstream or downstream of transcription start sites in a given cell subclass. Gene ontology analysis on differential regions was performed using the Genomic Regions Enrichment of Annotations Tool (GREAT, http://great.stanford.edu/public/html/)^135^.

### Bigwig generation for Paired-Tag sequencing data

BigWig files were generated from merged BAM files using the bamCoverage utility in deepTools^129^ with the parameters: *-e 100 -bs 100 --normalizeUsing RPKM*. The *smoothLength* parameter was set to 300, corresponding to three times the bin size, for H3K27ac, H3K4me1, and H3K27me3 signals; and to 10,000 for H3K9me3 to account for the broader enrichment profiles of this modification; no smoothing was performed for RNA signals.

### Comparative analysis of Chr-A and Chr-O cCREs

PhastCons conservation scores were computed using the computeMatrix function from deepTools^129^, with the parameters *scale-regions*, *--regionBodyLength 500 – missingDataAsZero --binSize 10*. This analysis was performed separately for Chr-A cCREs, Chr-O cCREs, and randomly shuffled genomic regions within each cell subclass. Conservation profiles were summarized by averaging PhastCons scores at each bin across all cell subclasses, yielding a representative conservation signal for each group of genomic regions. Random genomic regions were generated using the *shuffleBed* function from bedtools^134^ with the parameters *-chrom* and *-noOverlapping*. All cCREs identified from the snATAC-seq data were used as the foreground, and regions overlapping ENCODE mouse representative DNase-hypersensitive sites (rDHS) in the mm10 genome were excluded. Genomic distance analysis was performed only for pairs of cCREs located within the same chromosome. For such pairs, distances were defined as the genomic distance between their peak centers (summits). Distance distributions were calculated separately for Chr-A and Chr-O cCRE pairs within each cell subclass.

To assess the enhancer potential of Chr-A and Chr-O genomic regions, we used results from Allen Institute AAV-based enhancer assays as ground truth. Each tested genomic region was annotated as Chr-A-major or Chr-O-major within the corresponding cell types assayed in the Allen experiments, and these annotations were compared with experimental outcomes. For each Chr-A- or Chr-O-major group, true positives were defined as genomic regions showing positive or putative enhancer activity in the AAV assays, while false positives were regions with negative experimental results. Precision was calculated as the number of true positives divided by the sum of true and false positives. Recall was defined as the number of true positives divided by the total number of regions showing positive enhancer activity in the experiments. Specificity was defined as one minus the ratio of false positives to the product of the total number of genomic regions and the number of tested cell types. Chr-A regions identified by Paired-Tag and brain cCREs defined by snATAC-seq^17^ were also evaluated for enrichment of experimentally validated brain enhancers from the VISTA Enhancer Browser^63^ (https://enhancer.lbl.gov/vista/). Validated brain enhancers were defined as enhancers showing positive LacZ staining in the forebrain, midbrain, hindbrain, or neural tube. Enrichment of the overlap was quantified using odds ratios (OR) calculated from 2×2 contingency tables summarizing the number of regions that overlapped or did not overlap validated enhancers. Statistical significance was assessed using Fisher’s exact test.

### Motif enrichment analysis

Motif enrichment analysis was performed using HOMER (v5.1)^133^. Known motif enrichment was assessed using *findMotifsGenome.pl* on the mm10 genome, with input regions analyzed at their original lengths (*-size given*). A customized motif library derived from the JASPAR 2022 database (https://jaspar.elixir.no/) was used for known motif analysis. To reduce redundancy when summarizing motif enrichment across cell types, motifs with similar sequence patterns were merged into motif families using non-redundant transcription factor motif clustering (v2.0-beta) as described^123^.

### Analysis of transposable elements (TEs)

Chromatin coordinates and annotations of mouse transposable elements (TEs) were obtained from HOMER using the mm10 reference genome. Only TEs located on autosomal chromosomes (chromosomes 1–19) were included in downstream analyses. Information on the differential accessibility of TEs and predicted TE-promoter connections was adopted from our previous work^17^. TE-Chr-A elements were defined as TE-overlapping cCREs with Chr-A chromatin state annotation. TE subfamily-level activities were calculated using the average histone modification signals across all TEs belonging to that subfamily.

### Deep learning dataset assembly

In addition to processed Paired-Tag histone modification and RNA bigwig tracks, ATAC bigwig tracks were downloaded from https://catlas.org, and DNA methylation bigwig tracks were downloaded from https://data.nemoarchive.org/biccn/grant/u19_cemba/ecker/epigenome/cellgroup/mCseq3/mouse/processed/other/. Train test splits were downloaded from the borzoi manuscript by extracting fold 3 for the test set and fold 4 for the validation set, with the remaining folds used for training. Given the association between low-coverage data and extreme values in track quantification, we employed more aggressive data clipping than the Borzoi manuscript (see the git repo for details)^116^. Data was processed using the *hound_data* script from Baskerville (https://github.com/calico/baskerville) with one notable modification: rather than employing a fixed quantile at blacklist regions, we excluded them along-side genome gaps. Therefore, no blacklist regions were included in training or evaluation. Furthermore, we predicted values for a larger portion of the input sequence (512kb, rather than 196,608 bp). This was achieved by preserving the train/test annotation of contiguous genomic regions used to train Borzoi. As a result, the sequences making up individual train/test pairs are different, but data leakage is prevented.

### Borzoi model training

Models were trained using a modified version of the Baskerville repository, preserving the architecture of Borzoi^116^, but with modifications to the training procedure. Notably, we shifted from a loss-coupled L2 regularization to decoupled regularization^141^, and employed separate learning rates and decay parameters for convolutional and transformer layers. Two training settings were employed: a full fine-tuning setting where the model was initialized using the mouse model from the zeroth borzoi training replicate, and a from-scratch setting to compare the utility of epigenome tracks for guiding gene expression prediction performance. Given the different demands of the tasks, we employed one set of hyperparameters for fine-tuning, and another set was shared across all randomly initialized training tasks. Models trained from scratch were trained for 100 epochs, while the fine-tuned model was trained for 200 epochs. All models were trained using 8 A100s.

### Deep learning model evaluation

Before evaluation, we further filtered lower-quality tracks from the evaluation based on file size. All within-domain model evaluations were performed on unseen test sequences, using either hound_eval, or borzoi_test_genes. For causal variant prediction the TraitGym complex variant test set was downloaded from Hugging Face, consisting of 1,140 causal variants and nine variants with matched minor allele frequency (MAF) for each trait. To evaluate our model’s performance on brain-relevant traits, we subset to variants and matched negatives in TraitGym for the following traits: Alzheimer_LTFH, Glaucoma_Combined, Insomnia, Migraine_Self, Miserableness, Mood_Swings, Morning_Person, Smoking_Ever_Never, Worrier, Risk_Taking, and Suffer_from_Nerves. Trait identification performance was evaluated zero shot using the “L2 of L2” score as described^117^.

### External datasets

1. ENCODE mouse representative DNA hypersensitive site (rDHS) regions for mm10 are obtained from https://www.encodeproject.org/annotations/ENCSR672RVL/ in the ENCODE database.
2. ENCODE mouse candidate *cis-*regulatory elements (cCREs) for mm10 are obtained from https://screen.wenglab.org/ in the SCREEN database (version 4).
3. The bigwig format of PhastCons scores for multiple alignments of multiple species’ genomes to the mouse genome are downloaded from https://hgdownload.cse.ucsc.edu/goldenpath/mm10/phastCons60way/ maintained by the Genome Institute at UCSC.
4. ENCODE mouse blacklist version 2 for mm10 is downloaded from https://github.com/Boyle-Lab/Blacklist.
5. Mouse mm10 (GRCm 38) genome annotation information (version M25 or GRCm38.p6) was downloaded from https://www.gencodegenes.org/mouse/release_M25.html in GENCODE.

### Statistics

No statistical methods were used to predetermine sample sizes. There was no randomization of the samples, and investigators were not blinded to the specimens being investigated. Low-quality nuclei and potential doublets were excluded before downstream analysis as described before. Clustering of these single-nuclei RNA expression profiles or different histone modification profiles was performed in an unbiased manner. Peak callings on different histone modifications followed the procedure used in ENCODE in an unbiased manner.

## Supplementary Material

Supplement 1 Table S1. Paired-Tag oligo sequences.

Supplement 2 Table S2. Paired-Tag cell counts.

Supplement 3 Table S3. OPC to Oligo variable state regions.

Supplement 4 Table S4. Subclass-specific epigenomic features.

Supplement 5 Table S5. Subclass motif enrichment in Chr-A.

Supplement 6 Table S6. Sex-specific epigenomic and transcriptomic features.

Supplement 7 Table S7. Subclass-specific TE-Chr-A regions.

1 **Figure S1. Sample collection strategy, related to** Figure 1. A. Schematic overview of the mouse brain tissue dissection strategy. Mouse brains were sectioned into 600 μm thick coronal slices.
B. Brain regions analyzed in this study, their corresponding dissection region labels, and the matched brain region annotations registered in the Allen Brain Cell Atlas. The dissection region labels used here are consistent with those defined in prior work from the Center for Epigenomics of the Mouse Brain Atlas (CEMBA), generated as part of the Brain Research through Advancing Innovative Neurotechnologies (BRAIN) Initiative - Cell Census Network (BICCN).
C. Brain regions dissected from each coronal slice, annotated according to the Allen Brain Reference Atlas. The frontal view of slices 2–13 are shown, with the dissected region labels indicated on the left, and corresponding anatomical region annotations on the right.
D. Dot blot assays demonstrating the specificity and reactivity of antibodies used in this study against recombinant histone H3 carrying various histone modifications. H3K4me3 antibody and recombinant peptides were used here as control.
All brain maps shown in this figure were generated using coordinates from the Allen Mouse Brain Common Coordinate Framework (CCF) v3^46^.
**Figure S2. Quality control metrics, related to** Figure 1. A. Violin plots, from top to bottom, showing per-cell quality of number of RNA unique read counts, number of genes detected, percentage of mitochondrial reads, number of H3K27ac unique read counts, number of H3K27me3 unique read counts, number of H3K4me1 unique read counts, and number of H3K9me3 unique read counts, for each brain region analyzed.
B. Left, scatter plot showing the fraction of human and mouse RNA reads in each cell from the species-mixing experiment. Barcodes with fewer than 75% of reads from a single species were classified as doublets. Right, box plot showing the distribution and mean multiplet rate of the Paired-Tag dataset.
C. Heatmap showing the consensus scores between scRNA-seq subclasses and L5-level clusters derived from Paired-Tag RNA profiles.
D. Uniform manifold approximation project (UMAP) visualization of single cell Paired-Tag DNA profiles for each histone modification, from left to right: H3K27ac, H3K27me3, H3K4me1, and H3K9me3, colored by class labels. Normalized mutual information (NMI) and k-nearest neighbor label transfer accuracy values were used to evaluate clustering quality, with the Paired-Tag RNA modality achieving a NMI value of 0.7692 (cap).
**Figure S3. Cellular composition of the analyzed brain regions, related to** Figure 1. Bar plots depict the cellular composition of the nine analyzed brain regions. Subclasses with over 100 cells for each region are shown. Region-specific subclasses, defined as subclasses for which more than 75% of cells in the Paired-Tag dataset originate from a single dominant brain region, are shown in blue. Brain region schematics were generated using coordinates from the Allen Mouse Brain Common Coordinate Framework (CCF) v3^46^.
**Figure S4. ChromHMM modeling of chromatin states, related to** Figure 3. A. Heatmap showing the maximum Pearson’s correlation of each state in the full ChromHMM model (y-axis) with its best-matching state in each reduced model (x-axis).
B. Median Pearson correlation of all 24 states in each reduced ChromHMM model (x-axis). Models with 18 states and more showed a median Pearson correlation of > 99%.
C. Evaluation of k-means clustering using the ratio of between-cluster variance to total variance (Between SS/Total SS) across ChromHMM models with varying numbers of states. Models with 18 states and more showed a Between SS/Total SS of > 95%.
D. Emission probabilities of each chromatin mark for each state in the 18-state ChromHMM model.
E. Coverage heatmap showing the ATAC, H3K27ac, H3K27me3, H3K4me1, and H3K9me3 signals from the 005 L5 IT CTX Glut subclass across states in the 18-state ChromHMM model.
F. Box plot showing fraction of variable bases associated with each chromatin state across brain cell subclasses.
G. Box plots showing H3K27me3 signal on CpG islands stratified by Low (< 0.2), Medium (0.2~0.8) and High (> 0.8) mCG fraction in representative brain cell subclasses 005 L5 IT CTX Glut (left) and 327 Oligo NN (right). Statistical testing was performed using *t* test followed by Benjamini-Hochberg correction.
H. Box plot showing fraction of DNA non-CG methylation (mCH) associated with each chromatin state across neuronal brain cell subclasses.
I. Box plot showing fraction of DNA non-CG methylation (mCH) associated with each chromatin state across non-neuronal brain cell subclasses.
J. Bar plot showing aggregated genomic coverage of each chromatin state, along with genomic regions assigned functional chromatin state annotations, across all brain cell subclasses.
**Figure S5. Functional and epigenomic comparison of Chr-A and Chr-O, related to** Figure 4. A. Bar plot showing the fraction of human conserved cCREs in Chr-A and Chr-O.
B. Bar plot showing the fraction of non-conserved cCREs in Chr-A and Chr-O.
C. Box plot showing the fraction of differentially accessible regions (DAR) assigned as Chr-As and Chr-Os across different brain cell subclasses. Statistical significance was assessed using the Wilcoxon signed-rank test. ****, p-value < 0.0001.
D. Heatmap showing the dominant chromatin state annotation of validated enhancers from Allen Institute Enhancer (AiE) collection, with verified on-target cell-type-specific activity, across various relevant brain cell subclasses. These enhancers are stratified based on sequence conservation (presence of conserved sequence in the human genome), recall (whether Chr-A chromatin state annotation is detected in the validated target cell type), and target cell type (experimentally validated using AAV-enhancer reporter).
E. Bar plots showing the recall of Chr-A and Chr-O annotations in predicting validated cell type-specific enhancers from the Allen Institute Enhancer (AiE) AAV reporter collection. Recall is defined as the fraction of AAV reporter–validated enhancers that are annotated as Chr-A or Chr-O in the corresponding cell types.
F. Bar plots showing the specificity of Chr-A and Chr-O annotations in predicting validated cell type-specific enhancers from the Allen Institute Enhancer (AiE) AAV reporter collection. Specificity is defined as the fraction of cell types in which Allen AAV reporters tested negative and in which the corresponding genomic regions are not annotated as Chr-A or Chr-O.
G. Number of non-redundant TF motifs that are significantly enriched in Chr-A, Chr-Pr and Chr-O across selected brain cell subclasses. Non-redundant TF motifs were defined as described previously^123^.
H. Table summarizing the top five enriched TF motifs in Chr-A, Chr-Pr and Chr-O for brain cell subclasses 037 DG Glut and 334 Microglia NN.
I. Scatter plot showing the log2 Odds Ratio of TF motif enrichment in Chr-A compared with Chr-O across subclasses, with CTCF and CTCFL motifs highlighted.
J. Scatter plot showing the log2 Odds Ratio of TF motif enrichment in Chr-A compared with Chr-O across subclasses, with MEF2A, MEF2C and MEF2D motifs highlighted.
K. Heatmap showing enrichment of transcription factor binding motifs across all brain cell subclasses.
L. Coverage heatmaps of H3K27ac and H3K4me1 signals at differentially enriched H3K27ac peaks between anterior (HCa) and posterior (HCp) hippocampus in subclass 017 CA3 Glut neurons.
M. Coverage heatmaps of H3K27ac and H3K4me1 signals at differentially enriched H3K27ac peaks between HCa and HCp in subclass 037 DG Glut neurons.
**Figure S6. Cell-type-specific repressive features in the mouse brain, related to** Figure 5. A. Heatmap showing normalized H3K9me3 signal over subclass-enriched H3K9me3 features in selected CGE- and MGE-derived GABAergic neuronal subclasses.
B. Heatmap showing normalized H3K9me3 signal over subclass-depleted H3K9me3 features in selected CGE- and MGE-derived GABAergic neuronal subclasses.
C. Genome browser tracks showing ATAC, H3K27ac, H3K27me3, H3K4me1, H3K9me3, chromatin states, and RNA signals, for selected CGE- and MGE-derived GABAergic neuronal subclasses, at *Lhx6*, *Sox6* and *Fgf12* genomic loci.
D. Genome browser tracks showing RNA signals at *Fgf12* genomic loci for VIP, SNCG, LAMP5, PVALB and SST GABAergic neurons in mouse, marmoset, macaque, and human brains. Cell-type-resolved RNA-seq tracks of the brain from various species were generated using 10xGenomics Multiome from our previous work^11^.
**Figure S7. Sex-biased epigenomic features and transposable elements with regulatory signatures in the mouse brain, related to** Figure 6. A. Left, heatmap showing the number of differentially marked H3K27ac, H3K27me3, H3K4me1 and H3K9me3 peaks on autosomes across brain cell subclasses; Right, heatmap showing the number of sex-biased genes across brain cell subclasses, stratified by male-preferential and female-preferential genes on all chromosomes and autosomes only.
B-D. Left, genome browser tracks showing sex-differential H3K27ac signal at the *Xist* (B), *Kdm6a* (C), and *Kcnc2* (D) gene loci in 006 L4/5 IT CTX Glut subclass. Tracks are shown for male and female samples. Right, violin plots showing sex-differential expression of *Xist* (B), *Kdm6a* (C), and *Kcnc2* (D) in male and female cells of the same subclass. Statistical significance was assessed using Wilcoxon rank-sum test followed by Bonferroni correction.
E. Left, genome browser tracks showing sex-differential H3K27ac signal at the *St6galnac3* gene locus in 008 L2/3 IT ENT Glut subclass. Tracks are shown for male and female samples. Right, violin plot showing sex-differential expression of *St6galnac3* in male and female cells of the same subclass. Statistical significance was assessed using Wilcoxon rank-sum test followed by Bonferroni correction.
F. Left, genome browser tracks showing sex-differential H3K27ac signal at the *Sptbn2* gene locus in 014 LA-BLA-BMA-PA Glut subclass. Tracks are shown for male and female samples. Right, violin plot showing sex-differential expression of *Sptbn2* in male and female cells of the same subclass. Statistical significance was assessed using Wilcoxon rank-sum test followed by Bonferroni correction.
G. Left, genome browser tracks showing sex-differential H3K27ac signal at the *Sptbn2* gene locus in 014 LA-BLA-BMA-PA Glut subclass. Tracks are shown for male and female samples. Right, violin plot showing sex-differential expression of *Sptbn2* in male and female cells of the same subclass. Statistical significance was assessed using Wilcoxon rank-sum test followed by Bonferroni correction.
H. Box plot showing H3K27ac signals on TEs stratified by genomic context: TEs not overlapping brain cCRE (TE-BG), TEs overlapping brain cCREs (TE-cCRE), TEs overlapping Chr-As (TE-Chr-A), and TEs overlapping Chr-Os (TE-Chr-O). Statistical significance was assessed using two-sided *t* test with Benjamini-Hochberg correction.
I. Box plot showing H3K4me1 signals on TEs stratified by genomic context: TEs not overlapping brain cCRE (TE-BG), TEs overlapping brain cCREs (TE-cCRE), TEs overlapping Chr-As (TE-Chr-A), and TEs overlapping Chr-Os (TE-Chr-O). Statistical significance was assessed using two-sided *t* test with Benjamini-Hochberg correction.
J. Box plot showing the fraction of cCREs overlapping TEs in High-TE Glut subclasses compared with other, not High-TE subclasses. Statistical significance was accessed using Wilcoxon rank-sum test.
K. Box plot showing the fraction of Chr-As overlapping TEs in High-TE Glut subclasses compared with other, not High-TE subclasses. Statistical significance was accessed using Wilcoxon rank-sum test.
**Figure S8. Mouse brain Borzoi model, related to** Figure 7. A. Left, scatter plot showing the log10 number of cells in a cell type compared to the Pearson correlation between measurements and predictions in the test set. Right, scatter plot showing the log10 file size and the test set Pearson correlation for model predictions.
B. Scatter plot showing the log10 file size in bytes and the maximum RPKM value in a track for each histone modification track used in model training.
C. Scatter plot showing the log10 file size and the total RPKM values mapped to blacklist regions for each histone modification track used in training.
D. Hexbin plots showing the correlation between model predictions and measurements across subclasses and the coefficient of variation for each peak region in the test set. From left to right, plots correspond to H3K27ac, H3K27me3, H3K9me3, and ATAC.
E. Hexbin plot showing the Pearson correlation between predicted and measured average gene-level RPKM values for each gene in the test set.
F. Left, boxplots and histograms showing the distribution of mean RPKM across subclasses for each peak in the test set, stratified by modality. Right, boxplots and histograms showing the coefficients of variation for each peak in the test set.
G. Bar plot showing the area under the precision-recall curve (AUPRC) on TraitGym benchmark, comparing zero-shot causal variant detection performance of our model to Borzoi for all traits.

## Figures and Tables

**Table:** Key Resources Table

REAGENT or RESOURCE	SOURCE	IDENTIFIER
Antibodies		
Rabbit anti-H3K27ac	Abcam	Cat# ab4729; RRID: AB_2118291
Rabbit anti-H3K27me3	Abcam	Cat# ab195477; RRID: AB_2819023
Rabbit anti-H3K4me1	Abcam	Cat# ab8895; RRID: AB_306847
Rabbit anti-H3K9me3	Abcam	Cat# ab8898; RRID: AB_306848
Chemicals, peptides, and recombinant proteins		
Dulbecco’s Modified Eagle Medium	Thermo Fisher Scientific	Cat# 11995073
Fetal Bovine Serum	Omega Scientific	Cat# FB-02
Penicillin-Streptomycin-Glutamine (100X)	Thermo Fisher Scientific	Cat# 10378016
Trypsin-EDTA (0.25%)	Thermo Fisher Scientific	Cat# 25200056
Recombinant pA-Tn5	This paper	N/A
cOmplete^™^, EDTA-free Protease Inhibitor Cocktail	Roche	Cat# 11873580001
RNaseOUT	Invitrogen	Cat# 10777-019
SUPERase •In	Invitrogen	Cat# AM2694
Bovine Serum Albumin	Sigma Aldrich	Cat# A1595
Digitonin	Sigma Aldrich	Cat# 300410
Spermidine	Sigma Aldrich	Cat# 05292
Recombinant Albumin	NEB	Cat# B9200S
T4 DNA Ligase	NEB	Cat# M0202L
Proteinase K	NEB	Cat# P8107S
SPRIselect beads	Beckman Coulter	Cat# B23319
Terminal Transferase (TdT)	NEB	Cat# M0315L
SbfI-HF	NEB	Cat# R3642L
FokI	NEB	Cat# R0109L
NotI-HF	NEB	Cat# R3189L
KAPA HiFi HotStart PCR Kit	Roche	Cat# KK2502
NEBNext Ultra II Q5 Master Mix	NEB	Cat# M0544X
Maxima H minus Reverse Transcriptase	Thermo Fisher Scientific	Cat# EP0752
Critical commercial assays		
Nextera XT DNA Library Preparation Kit	Illumina	Cat# FC-131-1096
Qubit 1X dsDNA HS Assay	Invitrogen	Cat# Q33231
High Sensitivity D1000 ScreenTape	Agilent	Cat# 5067-5584
Deposited data		
Raw Paired-Tag sequencing data	This paper	RRID: SCR_016152; Collection IDs: nemo:col-wgpj7cd, nemo:col-xt1 ksye
Processed data portal	CATlas	RRID: SCR_018690; https://catlas.org/catlas/amb-pt
Deep learning model web portal	This paper	https://seqnn.org/
Mouse brain scRNA-seq data	Allen Brain Cell Atlas; Yao et al.^6^	https://brain-map.org/bkp/explore/abc-atlas
Mouse brain snATAC-seq data	Zu et al.^17^	https://www.catlas.org/catlas/dataset_resource.php?ID=wholemousebrain
Mouse brain snmC-seq and snm3C-seq	Liu et al.^16^	https://mousebrain.salk.edu/
Experimental models: Cell lines		
HeLa S3 cell line	ATCC	Cat# CCL-2.2; RRID: CVCL_0058
Experimental models: Organisms/strains		
Mouse: C57BL/6J	The Jackson Laboratory	Strain #: 000664; RRID: IMSR_JAX:000664
Oligonucleotides		
Paired-Tag primer oligos and barcodes	This paper	Table S1
Software and algorithms		
Paired-Tag processing pipeline	GitHub	https://github.com/zwang0715/Paired-Tag
Custom analysis scripts for Paired-Tag data	GitHub	https://github.com/beyondpie/amb_pairedtag
SnapATAC2 v2.8.0	Zhang et al.^47^	https://scverse.org/SnapATAC2/
Seurat v5.2	Hao et al.^124^	RRID: SCR_007322; https://satijalab.org/seurat/
scanpy v1.11.1	Wolf et al.^125^	RRID: SCR_018139
Harmony v1.2.0	Korsunsky et al.^126^	https://github.com/immunogenomics/harmony
ChromHMM v1.27	Ernst et al.^48^	RRID: SCR_018141; https://ernstlab.github.io/ChromHMM
ArchR v1.0.3	Granja et al.^127^	https://www.archrproject.com/
MACS3 v3.0.3	Zhang et al.^128^	https://macs3-project.github.io/MACS/
deepTools v3.5.6	Ramírez et al.^129^	https://deeptools.readthedocs.io/en/latest/
STAR v2.7.1a	Dobin et al.^130^	https://github.com/alexdobin/STAR
bowtie v1.3.1	Langmead et al.^131^	https://bowtie-bio.sourceforge.net/index.shtml
bowtie2 v2.5.1	Langmead et al.^132^	https://bowtie-bio.sourceforge.net/bowtie2/index.shtml
HOMER v5.1	Heinz et al.^133^	RRID: SCR_010881; v5.1; http://homer.ucsd.edu/homer/
bedtools v2.31.0	Quinlan^134^	https://bedtools.readthedocs.io/en/latest/
GREAT v4.0.4	McLean et al.^135^	http://great.stanford.edu/public/html/
scikit-learn v1.8.0	Pedregosa et al.^136^	https://scikit-learn.org/stable/
umap	McInnes et al.^137^	https://umap-learn.readthedocs.io/en/latest/
borzoi	Linder et al.^116^	https://github.com/calico/borzoi

## HIGHLIGHTS

- A single-cell epigenome atlas of transcription and four histone modifications across 2.5M mouse brain cells.
- Multi-modal integration functionally annotates ~81% of the adult mouse brain regulatory genome
- Cell-type-resolved chromatin landscapes reveal regulatory programs mediated by enhancers, Polycomb and H3K9me3
- Deep learning models predict cell-type-specific epigenomic features and gene expression from DNA sequence
