# Supplementary figures and images for: Integrative Single-Cell Epigenomic Atlas Annotates the Regulatory Genome of the Adult Mouse Brain
Source: bioRxiv. 2026 Feb 7:2026.02.07.704075. Preprint. [Version 1] doi: 10.64898/2026.02.07.704075 (PMC12889674; doi:10.64898/2026.02.07.704075)

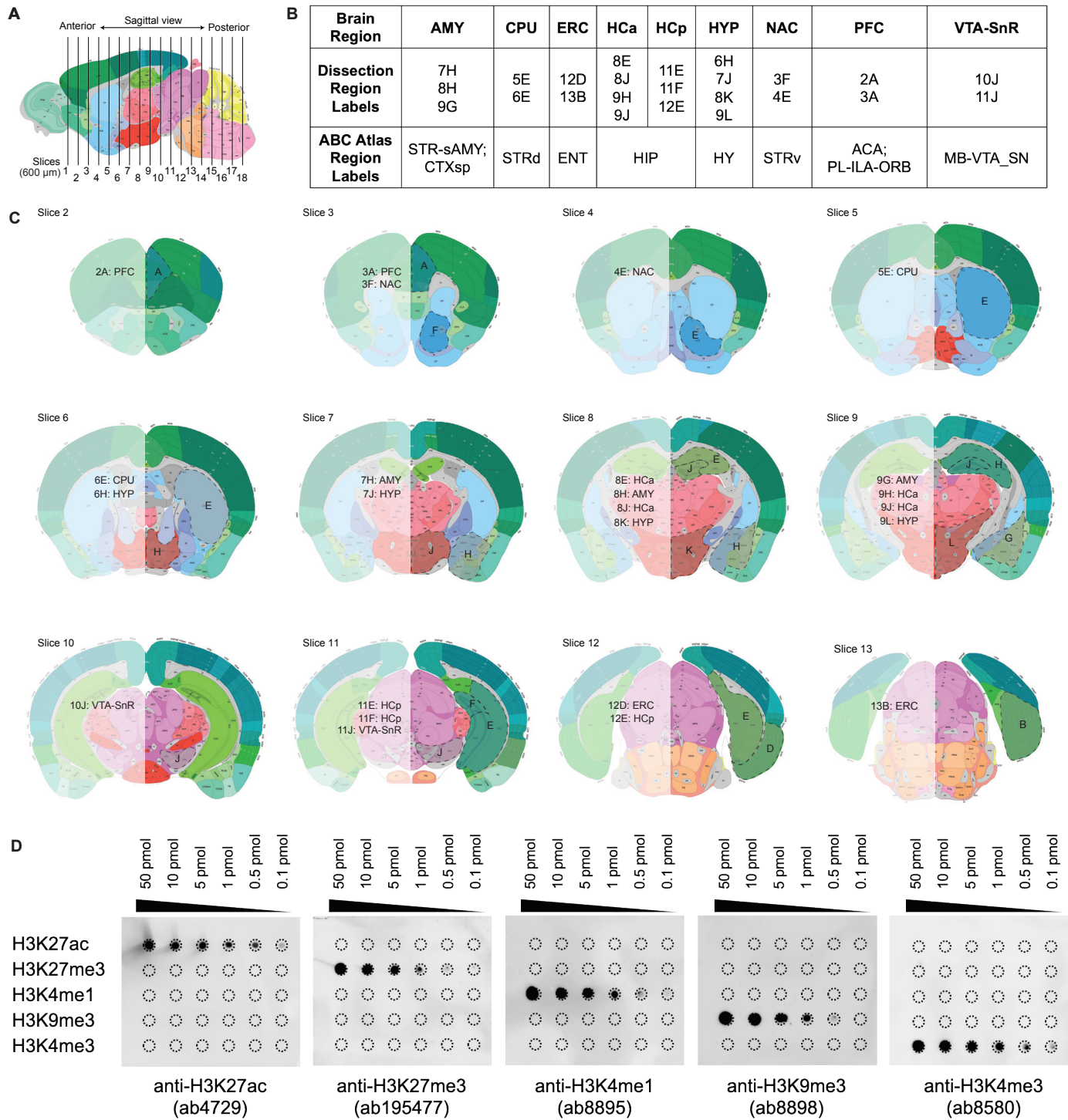

Figure S1

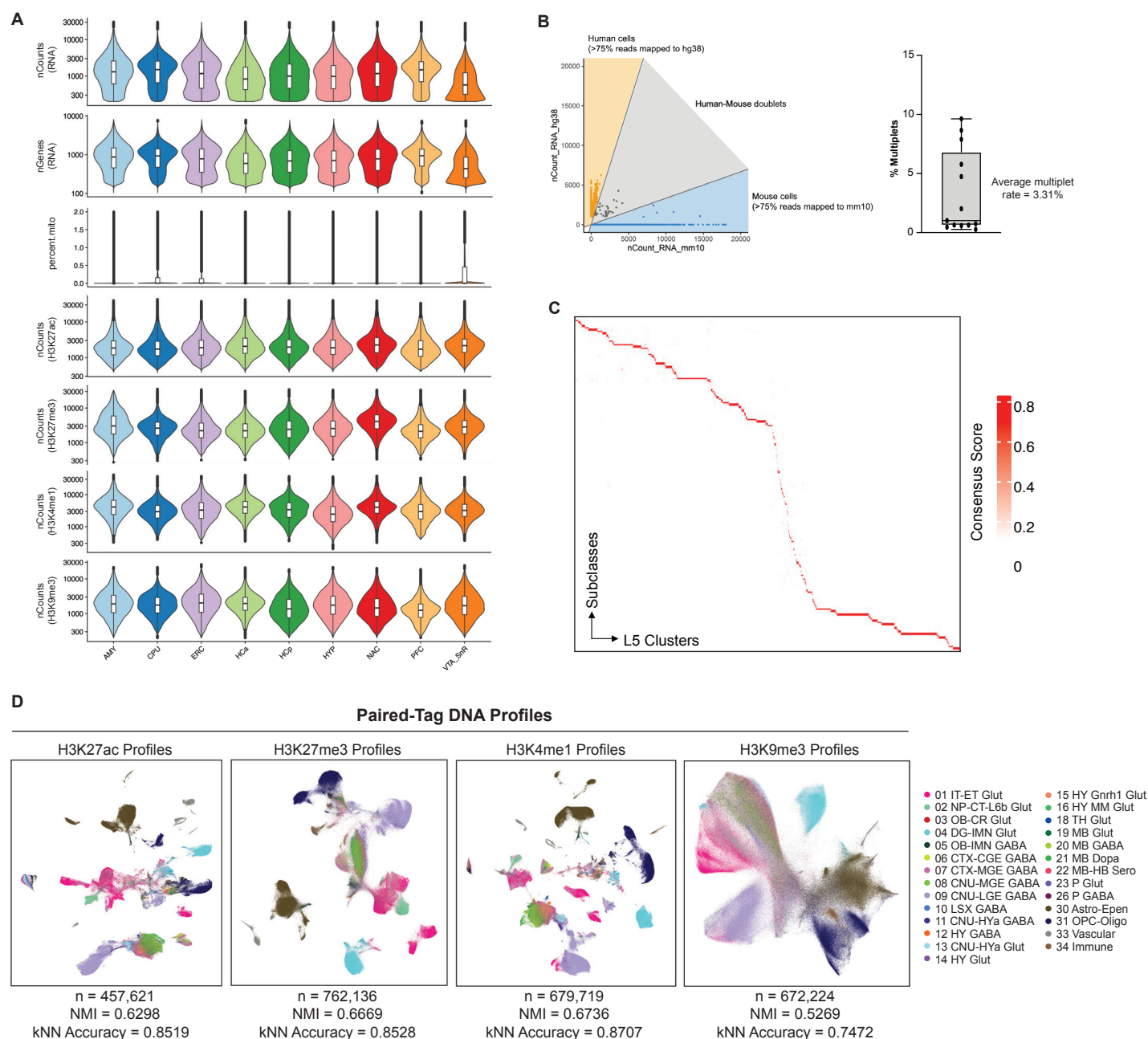

Figure S2

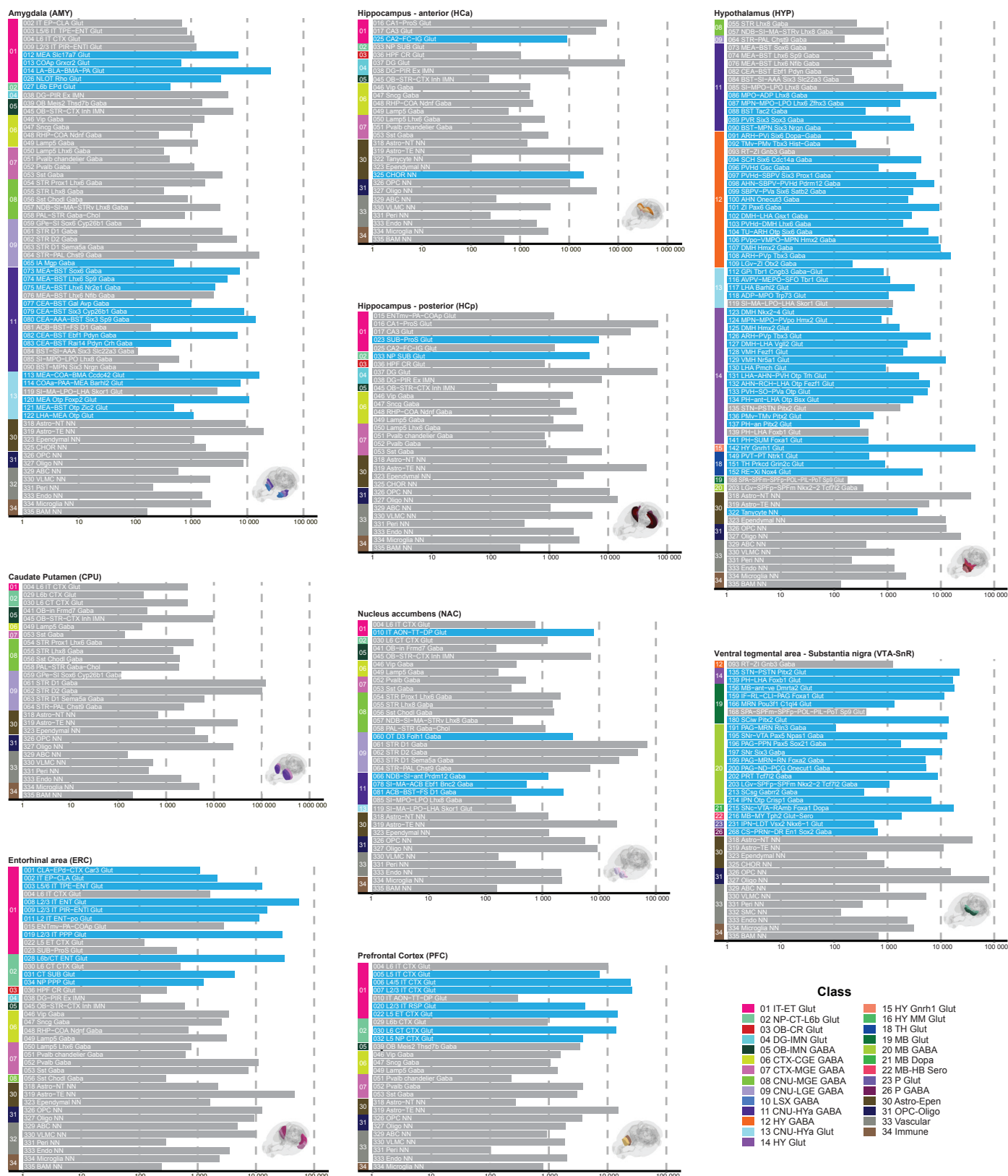

Figure S3

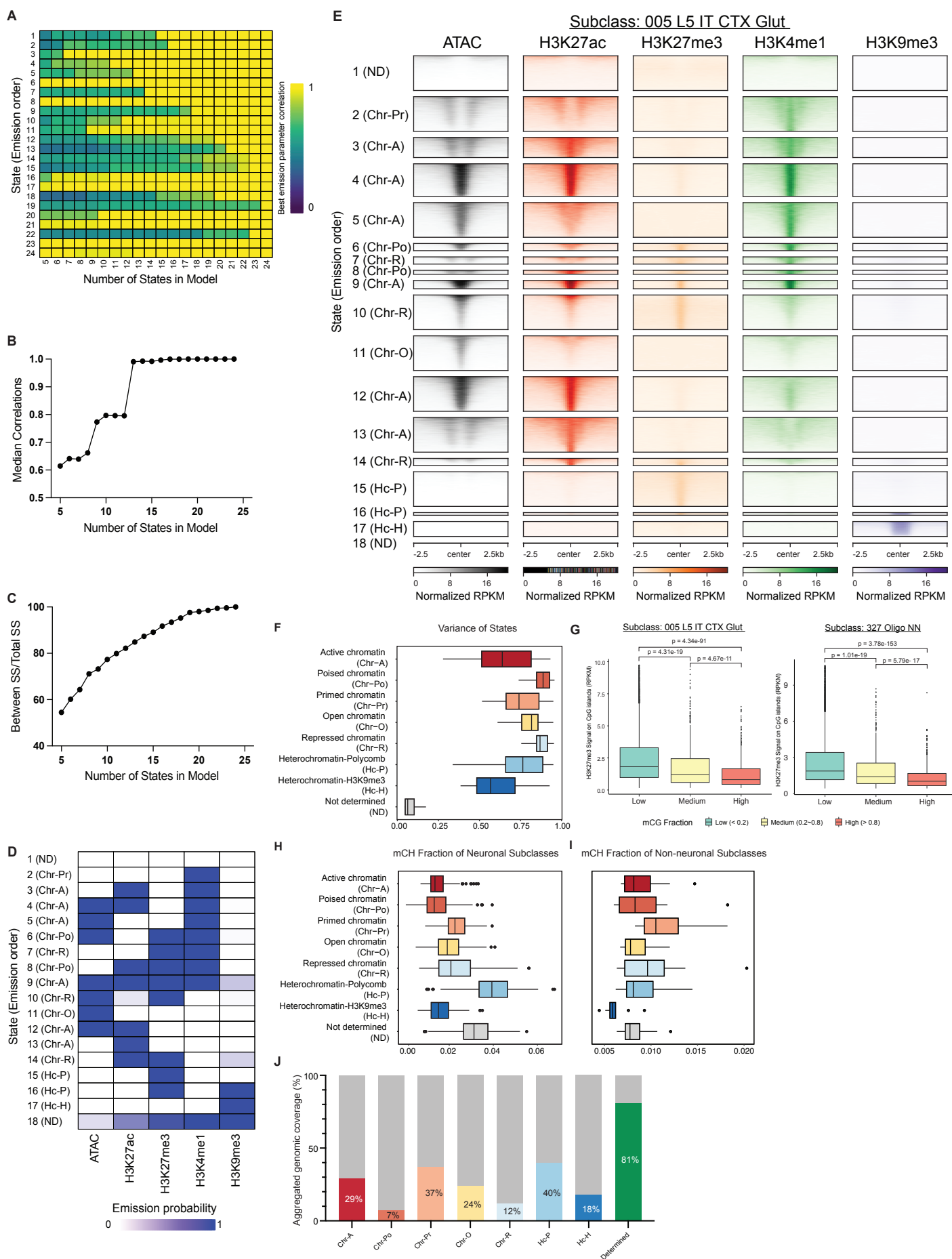

Figure S4

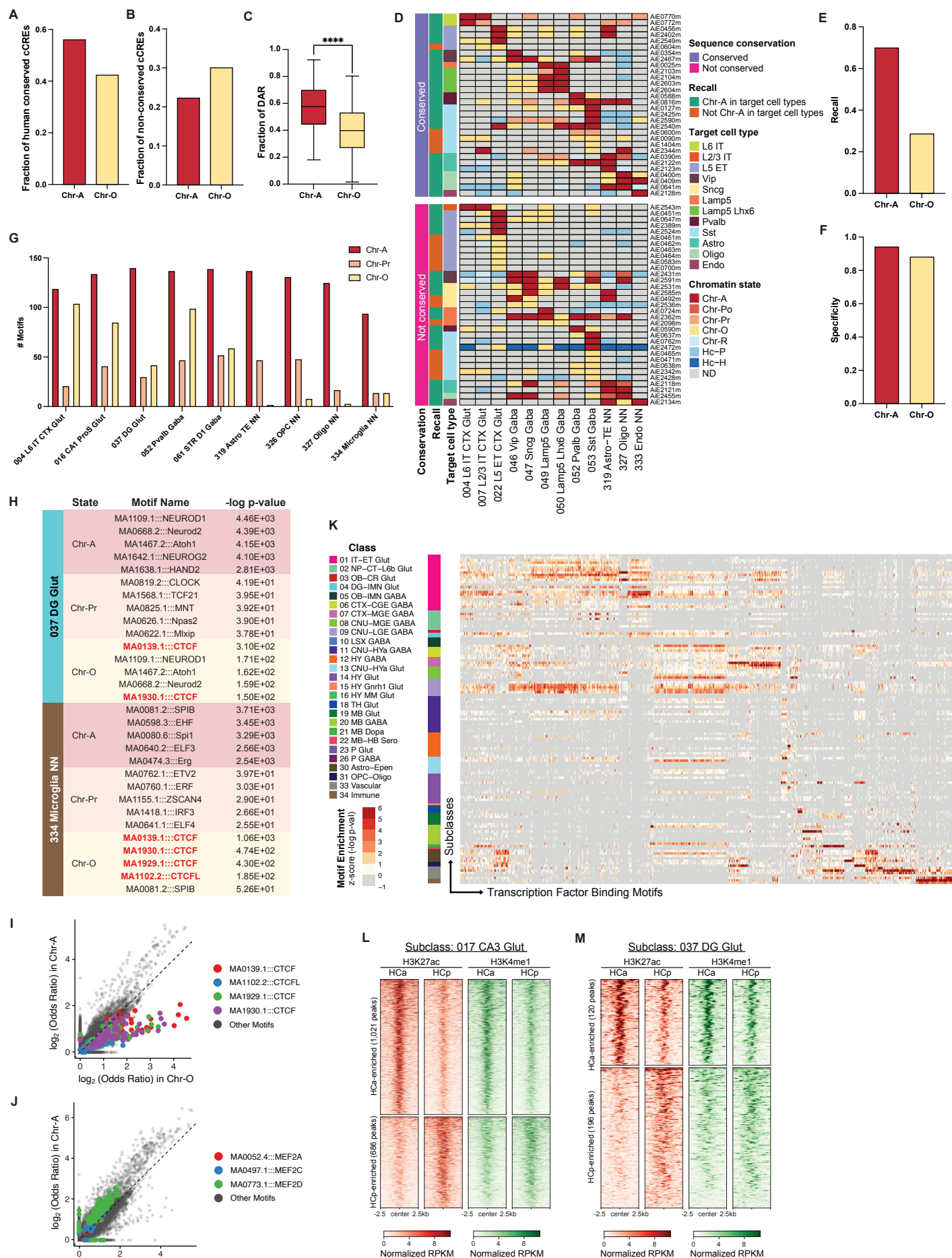

Figure S5

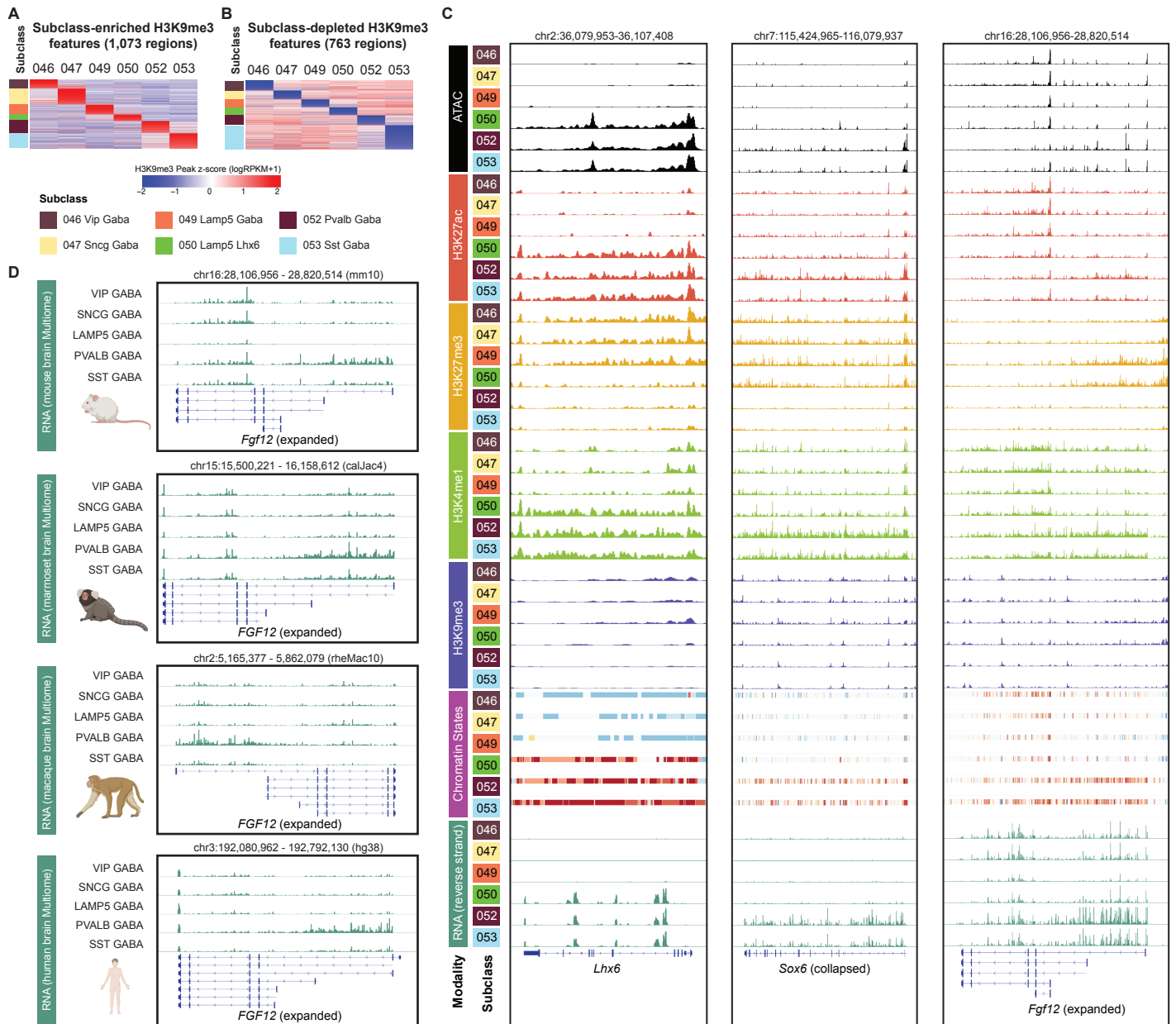

Figure S6

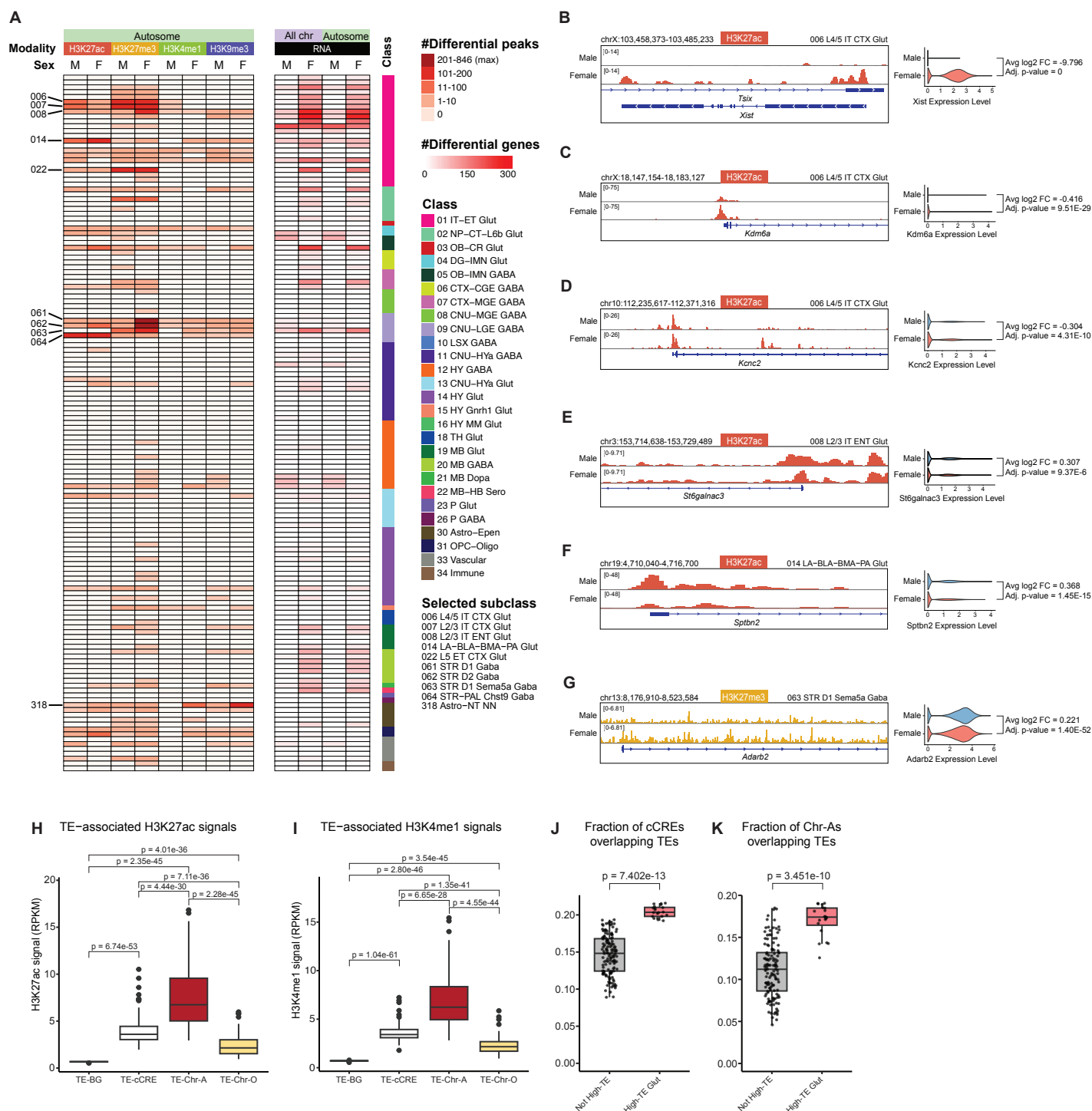

Figure S7

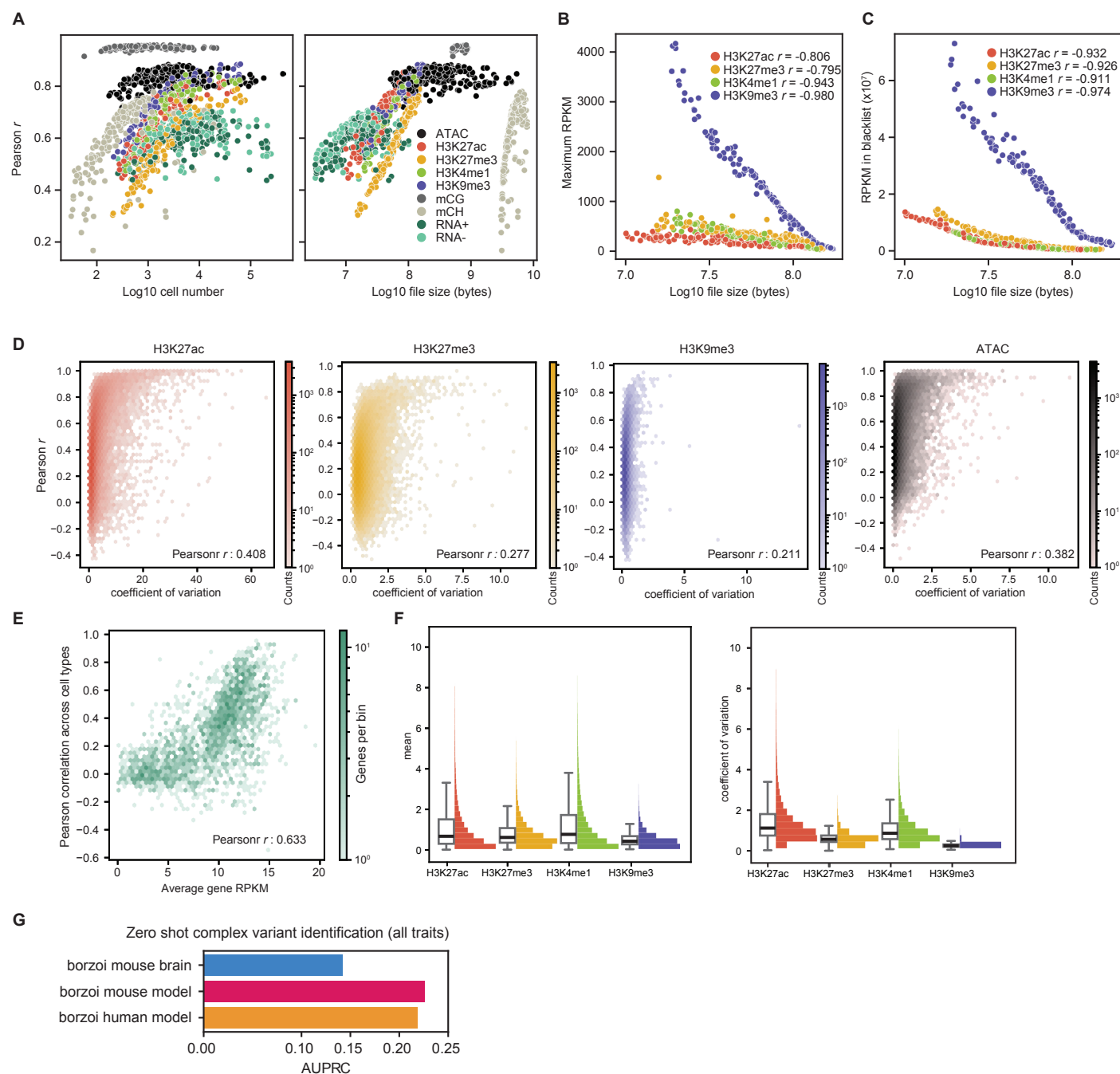

**Figure S8**

Supplement: 1 — Figure S1. Sample collection strategy, related to Figure 1. A. Schematic overview of the mouse brain tissue dissection strategy. Mouse brains were sectioned into 600 μm thick coronal slices. B. Brain regions analyzed in this study, their corresponding dissection region labels, and the matched brain region annotations registered in the Allen Brain Cell Atlas. The dissection region labels used here are consistent with those defined in prior work from the Center for Epigenomics of the Mouse Brain Atlas (CEMBA), generated as part of the Brain Research through Advancing Innovative Neurotechnologies (BRAIN) Initiative - Cell Census Network (BICCN). C. Brain regions dissected from each coronal slice, annotated according to the Allen Brain Reference Atlas. The frontal view of slices 2–13 are shown, with the dissected region labels indicated on the left, and corresponding anatomical region annotations on the right. D. Dot blot assays demonstrating the specificity and reactivity of antibodies used in this study against recombinant histone H3 carrying various histone modifications. H3K4me3 antibody and recombinant peptides were used here as control. All brain maps shown in this figure were generated using coordinates from the Allen Mouse Brain Common Coordinate Framework (CCF) v346. Figure S2. Quality control metrics, related to Figure 1. A. Violin plots, from top to bottom, showing per-cell quality of number of RNA unique read counts, number of genes detected, percentage of mitochondrial reads, number of H3K27ac unique read counts, number of H3K27me3 unique read counts, number of H3K4me1 unique read counts, and number of H3K9me3 unique read counts, for each brain region analyzed. B. Left, scatter plot showing the fraction of human and mouse RNA reads in each cell from the species-mixing experiment. Barcodes with fewer than 75% of reads from a single species were classified as doublets. Right, box plot showing the distribution and mean multiplet rate of the Paired-Tag dataset. [file NIHPP2026.02.07.704075V1-supplement-1.pdf]
